# Reactivity of a triamidoamine terminal uranium(vi)-nitride with 3d-transition metal metallocenes†

**DOI:** 10.1039/d4cc03846k

**Published:** 2024-08-15

**Authors:** John A. Seed, Peter A. Cleaves, Georgina R. Hatton, David M. King, Floriana Tuna, Ashley J. Wooles, Nicholas F. Chilton, Stephen T. Liddle

**Affiliations:** a Department of Chemistry, The University of Manchester Oxford Road Manchester M13 9PL UK steve.liddle@manchester.ac.uk; b School of Chemistry, The University of Nottingham University Park Nottingham NG7 2RD UK; c Department of Chemistry and Photon Science Institute, The University of Manchester Oxford Road Manchester M13 9PL UK; d Research School of Chemistry, The Australian National University Sullivans Creek Road Canberra ACT 2601 Australia nicholas.chilton@anu.edu.au

## Abstract

Reactions between [(Tren^TIPS^)U^VI^≡N] (1, Tren^TIPS^ = {N(CH_2_CH_2_NSiPr^i^_3_)_3_}^3−^) and [M^II^(η^5^-C_5_R_5_)_2_] (M/R = Cr/H, Mn/H, Fe/H, Ni/H) were intractable, but M/R = Co/H or Co/Me afforded [(Tren^TIPS^)U^V^=N-(η^1^:η^4^-C_5_H_5_)Co^I^(η^5^-C_5_H_5_)] (2) and [(Tren^TIPS^)U^IV^–NH_2_] (3), respectively. For M/R = V/H [(Tren^TIPS^)U^IV^–N=V^IV^(η^5^-C_5_H_5_)_2_] (4), was isolated. Complexes 2–4 evidence one-/two-electron uranium reductions, nucleophilic nitrides, and partial N-atom transfer.

## Abstract 2

The reactions of a uranium(vi)-nitride with 3d-transition metal metallocenes have been examined.

## Graphical abstract

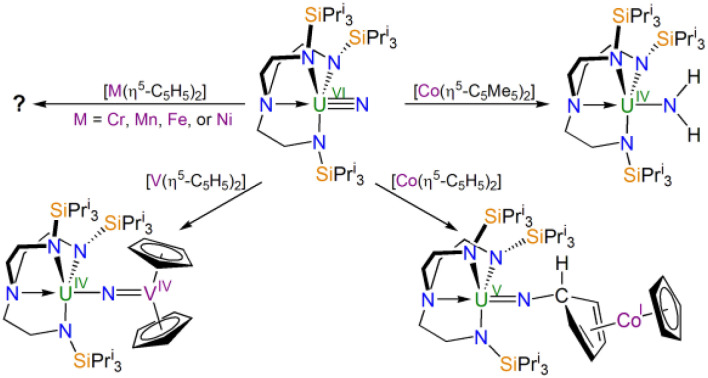

In recent years molecular uranium-nitrides have attracted burgeoning attention due to their importance as actinide electronic structure benchmarks and in small molecule activations.^1–4^ The search for isolable terminal uranium-nitrides was accomplished by some of us just over a decade ago, first with [Na(12C_4_)_2_][(Tren^TIPS^)U^V^≡N] (Tren^TIPS^ = {N(CH_2_CH_2_NSiPr^i^_3_)_3_}^3−^; 12C_4_ = 12-crown-4 ether)^5^ in 2012 and then [(Tren^TIPS^)U^VI^≡N] (1) in 2013.^6^ The Tren^TIPS^ ligand has proven to be a ‘privileged’ ancillary ligand for terminal uranium-nitrides,^7–10^ and indeed the only other ligand class to have supported an isolable terminal uranium-nitride linkage is the siloxide ligand (Bu^*t*^O)_3_SiO^1−^ used by Mazzanti.^11^ In addition to terminal uranium-nitrides, a variety of low- (two-) coordinate bridging uranium-nitrides are now known, including U≡NAM (AM = Li, Na, K, Rb, Cs),^6,8,12,13^ U=N=An (An = U, Th),^13–28^ and U≡N–M complexes (M = Mo, Rh, Ir, Mo).^29,30^ The latter remain few in number, likely largely reflecting the limited synthetic methodologies available for constructing such linkages: M = Mo was accessed by partial nitride transfer from Mo to U,^29^ and M = Rh and Ir compounds were made by photolysis of azido precursors.^30^ We decided to examine the potential of 1 to construct heterobimetallic nitride-bridged complexes since it already has a terminal,^5–10^ nucleophilic nitride installed at uranium which could in principle simplify its use in synthesis.

Here we report on our findings, where we have examined the reactivity of 1 towards 3d transition metal metallocenes [M^II^(η^5^-C_5_R_5_)_2_] (M/R = V/H, Cr/H, Mn/H, Fe/H, Co/H, Co/Me, Ni/H). The reactions with M = Cr, Mn, Fe, and Ni appeared to proceed but proved intractable. However, reactions with M/R = Co/H, Co/Me, and V/H produced isolable derivatives that evidence one- and two-electron reductions of uranium, nucleophilic nitrides, and partial N-atom transfer.

In separate reactions, Scheme 1, mixing [(Tren^TIPS^)U^VI^≡N] (1) with [M^II^(η^5^-C_5_H_5_)_2_] (M = Cr, Mn, Fe, Ni) in cold (−78 °C) toluene afforded, after solvent was removed, crude brown solids. However, in all cases no products could be isolated cleanly. ^1^H NMR spectroscopy revealed numerous paramagnetically shifted resonances (up to 66 ppm wide range of resonances, Fig. S1–S4, ESI†) and hence the product identities and/or extent of decomposition is unclear.

**Scheme 1:**
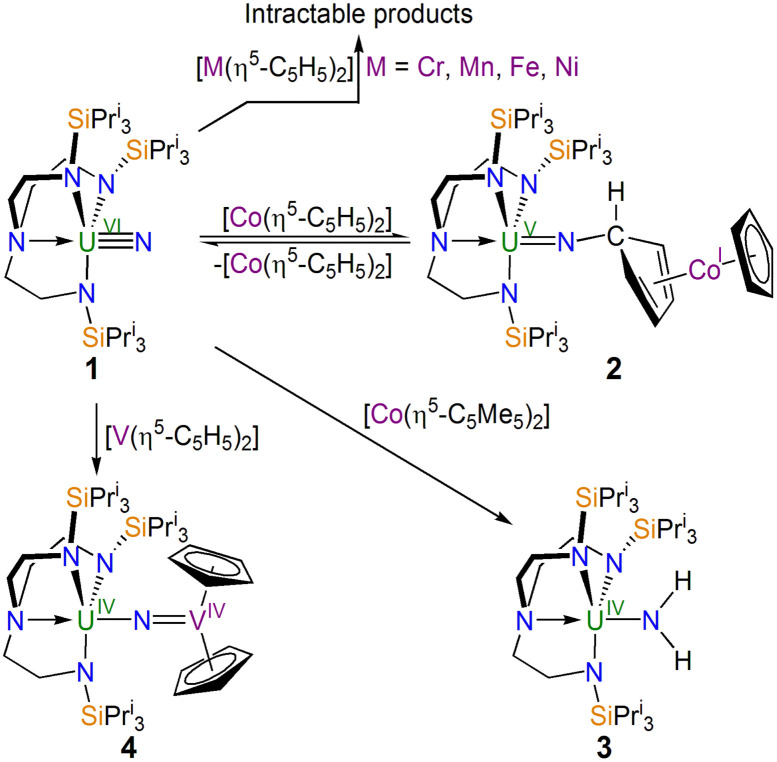
Synthesis of 2–4 from 1 and intractable reaction outcomes. The by-products are either not known or are not shown for clarity.

In contrast to the reactions between 1 and M = Cr, Mn, Fe, and Ni, with M = Co an identifiable product could be obtained, Scheme 1. Specifically, treating 1 with nineteen valence electron [Co^II^(η^5^-C_5_H_5_)_2_] afforded the uranium(v)-imido complex [(Tren^TIPS^)U^V^=N-(η^1^:η^4^-C_5_H_5_)Co^I^(η^5^-C_5_H_5_)] (2) as red crystals. However, 2 co-crystallises with variable quantities of 1 and [Co^II^(η^5^-C_5_H_5_)_2_] (Fig. S5 and S6, ESI†). Indeed, a variable-temperature ^1^H NMR study (Fig. S7, ESI†) revealed the dominance of 2 at low temperature (−60 °C) and a greater proportion of 1/[Co^II^(η^5^-C_5_H_5_)_2_] at higher temperature (25 °C), and hence 2 is in equilibrium with 1 and [Co^II^(η^5^-C_5_H_5_)_2_]. Whilst the optimal practical ratio for the reaction was found to be two equiv. of [Co^II^(η^5^-C_5_H_5_)_2_] to 1 we could only ever isolate 2 as a mixture (A) co-crystallised with 1 and [Co^II^(η^5^-C_5_H_5_)_2_]. Although the [Co^II^(η^5^-C_5_H_5_)_2_] can be sublimed out of A, when redissolved in addition to 2 resonances for 1 and [Co^II^(η^5^-C_5_H_5_)_2_] are still observed in the resulting ^1^H NMR spectrum demonstrating an immutable equilibrium.

Nucleophilic attack of eighteen valence electron [Co^III^(η^5^-C_5_H_5_)_2_]^+^ is known,^31^ and whilst a radical reaction cannot be discounted the radical chemistry of 1 is quite slow in the absence of strong light,^6^ so we propose that [Co^II^(η^5^-C_5_H_5_)_2_] (*E*^0′^ = ∼−1.32 V *vs.* Fc)^32^ initially reduces 1 to give “[Co^III^(η^5^-C_5_H_5_)_2_]^+^[(Tren^TIPS^)U^V^≡N]^−^”, and then nucleophilic attack of a cyclopentadienyl ring by the nitride occurs. The nucleophilic attack rehybridises one of the cyclopentadienyl carbon atoms from sp^2^ to sp^3^, formally forming a Co^I^-cyclopentadiene unit, hence retaining an eighteen valence electron cobalt moiety.

Given the issue in isolating 2, its characterisation was probed using A as far as was reasonably practicable. The ^1^H NMR spectrum of A exhibits resonances for 2 over the range 23.5 to −4.2 ppm (Fig. S5 and S6, ESI†). Of most salience, in addition to one cyclopentadienyl ring resonance of 5H (9.7 ppm) two pairs of 2H each for the η^4^-diene portion of the cyclopentadiene ring are located at 17.9 and 10.6 ppm, and the H-atom residing on the ring sp^3^ C-atom resonates at −1.5 ppm. We recorded the UV/Vis/NIR spectra of 1 and [Co^II^(η^5^-C_5_H_5_)_2_] and then subtracted them from the corresponding spectrum of A to unambiguously identify absorptions that correspond to 2 (Fig. S12–S14, ESI†). Of most interest is the near infrared region, where four absorptions (*ε* = ∼10–30 M^−1^ cm^−1^) are found at ∼6000, ∼7100, ∼9000, and ∼10 600 cm^−1^ which represent ^2^Γ_4_ to ^2^Γ_4_, ^2^Γ_4_, ^2^Γ_4_, and ^1^Γ_5_ + ^1^Γ_6_ absorptions, respectively, that are characteristic of uranium(v) in *C*_3v_ symmetry.^33^

The solid-state structure of 2 was determined, Fig. 1, confirming its formulation and also *exo*-attack by the nitride. The U1–N5 distance of 1.925(3) Å is longer than the terminal U^VI^≡N distance of 1.799(7) Å in 1 and group 1 capped and terminal (Tren^TIPS^)U^V^≡N distances (1.801(7)–1.840(3) Å),^5,6,8,12^ slightly shorter than (Tren^TIPS^)U^V^=NR distances (∼1.95 Å),^6^ though similar to [(Tren^TIPS^)U^V^≡NM]_2_ (AM = Li, Na, K, Rb, Cs) U^V^≡N distances (1.833(4)–1.929(6) Å).^5,8^ The N5–C34, C34–C35, and C34–C38 distances of 1.475(5), 1.516(5), and 1.525(15) Å are consistent with N–C and C–C single bonds, and the presence of the Co-bound diene is reflected by C35–C36, C36–C37, and C37–C38 distances of 1.414(5), 1.422(5), and 1.414(6) Å. All other distances in 2 are as anticipated. Overall, the metrical data are consistent with 2 being a uranium(v)-imido complex consistent with the UV/Vis/NIR data.

**Fig. 1:**
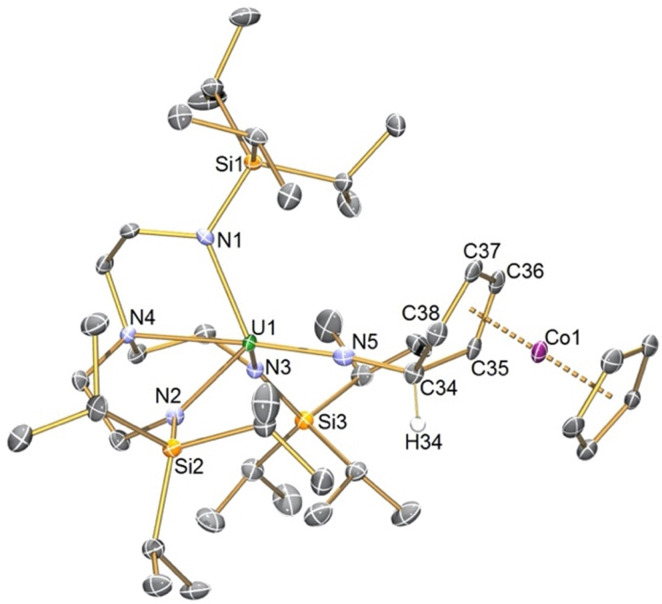
Molecular structure of 2 with selective labelling at 120 K and displacement ellipsoids at 50%. Hydrogen atoms except for H34 are omitted for clarity.

Density functional theory (DFT) calculations on 2 (Fig. S26, S27 and Tables S1–S3, S6, ESI†) reveal a somewhat delocalised picture, however the principal UN- and Co-related bonding combinations could be identified and natural bond orbital (NBO) and natural localised molecular orbital (NLMO) analyses identify the σ^2^π^4^ bonding motif of the imido (Fig. S27, ESI†). The computed charges and spin densities are consistent with U^V^/Co^I^. The U–N_imido_ Nalewajski–Mrozek bond order is 2.73, and quantum theory of atoms-in-molecules (QTAIM) analysis reveals a UN 3,−1-bond critical point with a *ρ* value of 0.18 that is typical of a uranium(v)-imido complex.^6^

Noting the reaction between 1 and [Co^II^(η^5^-C_5_H_5_)_2_], we examined the analogous reaction with [Co^II^(η^5^-C_5_Me_5_)_2_], Scheme 1: [Co^II^(η^5^-C_5_Me_5_)_2_] is a stronger reducing agent (∼−1.93 V *vs.* Fc)^32^ compared to [Co^II^(η^5^-C_5_H_5_)_2_], meaning an excess of Co-reagent would be less likely to be needed possibly simplifying purification, and the former is sterically more congested which may impede *exo*-addition. Thus, we treated 1 with one equiv. of [Co^II^(η^5^-C_5_Me_5_)_2_], and after work-up and recrystallisation isolated the previously reported emerald green amido complex [(Tren^TIPS^)U^IV^–NH_2_] (3).^6,9,34^

The formation of 3 seems at first surprising, but can be rationalised. Assuming that the reaction proceeds by U-reduction to form “[Co^III^(η^5^-C_5_Me_5_)_2_]^+^[(Tren^TIPS^)U^V^≡N]^−^”, protonation to give [(Tren^TIPS^)U^V^=NH] could occur, and it is known that oxidation of [(Tren^TIPS^)U^IV^=NH]^−^ results in the formation of 3 and 1*via* disproportionation of [(Tren^TIPS^)U^V^=NH].^34^ Alternatively, given the reducing nature of [Co^II^(η^5^-C_5_Me_5_)_2_], it could be that double reduction of 1 occurs to give “[(Tren^TIPS^)U^IV^≡N]^2−^”, which would be very reactive. Indeed, the closely related complex [(Tren^TIPS^)U^IV^≡NLi_2_]_2_ contains bridging nitrides and of all the group 1 cations is only stable with Li because of the highly polarised nature of the U^IV^≡N linkage with substantial destabilising charge accumulation at the nitride.^35^ We note that (C_5_Me_5_)^1−^ can provide protons *via* tuck-in/tuck-over complexes,^36^ and that [Co^II^(η^5^-C_5_Me_5_)_2_] can act as a H-atom shuttle,^37^ and either process could potentially expedite the formation of 3 from 1.

Since the exact nature of divalent group 4 metallocenes can be ambiguous, we lastly examined the reaction of 1 with [V^II^(η^5^-C_5_H_5_)_2_], Scheme 1. Accordingly, a 1 : 1 mixture was stirred in toluene, and after work-up the red complex formulated as [(Tren^TIPS^)U^IV^–N=V^IV^(η^5^-C_5_H_5_)_2_] (4) was isolated in 82% yield.

The solid-state structure of 4 confirms its gross formulation, Fig. 2. The U1–N5 distance of 2.261(9) Å is much longer than the U–N distances in 1^6^ and 2 but similar to the U–N amido distance in 3 (2.228(4) Å).^6,34^ Whilst the U1–N5 distance in 4 would be incompatible with U/V oxidation state combinations of VI/II and V/III, IV/IV and III/V were possible. However, the U–N_amide_ and –N_amine_ distances of 2.261(9)–2.285(8) and 2.674(10) Å are suggestive of U^IV^ over U^III^.^38^ The V1–N5 distance was found to be 1.680(9) Å, which compares to a V–N distance of 1.665 Å in [Me_3_SiN=V^IV^(η^5^-C_5_H_5_)_2_].^39^ Hence, 4 can be considered to result from partial N-atom transfer from U to V.

**Fig. 2:**
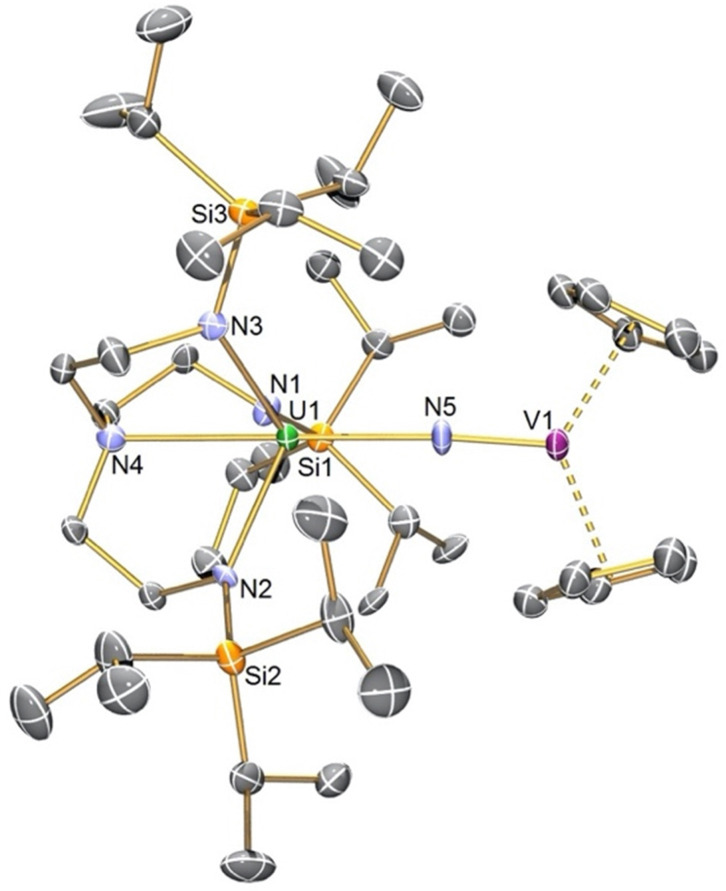
Molecular structure of 4 with selective labelling at 120 K and displacement ellipsoids at 50%. Hydrogen atoms are omitted for clarity.

The ^1^H NMR spectrum of 4 (Fig. S8, ESI†) covers the range 72.5 to −6.6 ppm; the resonance at 72.5 ppm corresponds to the vanadocene moiety, with the remaining resonances spanning 32.5 to −6.6 ppm which is qualitatively consistent with U^IV^. The ^29^Si{^1^H} NMR spectrum of 4 (Fig. S9, ESI†) exhibits a resonance at −76.2 ppm which falls in the range of U^IV^ complexes.^40^

The UV/Vis/NIR spectrum of 4 (Fig. S15–S17, ESI†) above 22 500 cm^−1^ is dominated by charge-transfer bands and a prominent absorption is found at ∼18 000 cm^−1^ (*ε* = ∼1500 M^−1^ cm^−1^). Below 15 000 cm^−1^ the spectrum evidences weak (*ε* = ∼30–70 M^−1^ cm^−1^) f–f absorptions. The NIR region has the appearance of U^IV^,^3^ but we could not completely rule out the broad feature at 18 000 cm^−1^ being f–d transitions of U^III^ rather than d–d transitions of V.^3^

Given the potential ambiguity of the U/V oxidation states in 4 we turned to quantum chemical calculations. However, DFT geometry optimisation always led to the U–N and V–N distances being too short and long, respectively (both ∼1.95 Å). Therefore, to first resolve the oxidation state question we turned to state-averaged complete active space self-consistent field (SA-CASSCF) calculations using the unoptimised crystal structure of 4 with an active space of 3 electrons in 12 orbitals (3d and 5f) examining low spin (*S*_tot_ = 1/2, 4′) and high spin (*S*_tot_ = 3/2, 4′′) multiplicities (see ESI† for details). The ground state is found to be dominated by U^IV^ (5f^2^) and V^IV^ (3d^1^) configurations, consistent with the foregoing characterisation data overall. Interestingly, the ground Kramers doublet after spin–orbit coupling is dominated by *S*_tot_ = 1/2 states, suggesting that there is an antiferromagnetic interaction between the V^IV^ and U^IV^ ions. Furthermore, the calculations show that there significant covalency and crystal field splitting of the 3d- and 5f-orbitals quenching the orbital angular momentum of 4 (see ESI†).

To confirm the SA-CASSCF findings, we collected variable-temperature SQUID magnetometry data on powdered 4 in an external 0.1 T field (Fig. S18, ESI†). The effective magnetic moment of 4 is 2.71*μ*_B_ at 300 K and this decreases steadily until at 8 K (1.21*μ*_B_) when it drops more rapidly reaching 0.42*μ*_B_ at 1.8 K. The magnetic moment for 4 falls far more quickly with decreasing temperature than for isolated U^IV^ in [{(Me_3_Si)_2_N}_3_U^IV^=E]^−^ (E = O, NSiMe_3_),^41^ suggesting antiferromagnetic coupling which is also implied by a maximum in the *χ*_M_*vs. T* plot of 4 at 4.8 K. The magnetisation at 1.8 K and 7 T (Fig. S21, ESI†) of 0.24*μ*_B_ mol^−1^ is also far smaller than the sum of an isolated [{(Me_3_Si)_2_N}_3_U^IV^=E]^−^ and a free *S* = 1/2,^41^ again reflecting the presence of U–V magnetic exchange.

The X-band EPR spectrum of powdered 4 (Fig. S22, ESI†) exhibits an eight line spectrum (^51^V, *I* = 7/2) with *g* = 1.971 (*A*_*x,y*_(^51^V) = 35 MHz and *A*_*z*_(^51^V) = 220 MHz). However, this is incompatible with the low-temperature SQUID magnetometry data which indicates a *S*_eff_ = 1/2 state with *g* ∼ 0.7. Indeed, the SA-CASSCF results suggest a strongly axial ground doublet state (Table S5, ESI†), and previous work has continuously highlighted the effective high-symmetry behaviour of pseudo-*C*_3_ U^IV^ fragments.^6,8,41^ Taking the data together, we suggest that 4 is actually EPR silent, and that due to the high sensitivity of EPR a trace impurity has been observed instead. We suggest that this is most likely [HN=V^IV^(η^5^-C_5_H_5_)_2_] given the similarity of our EPR data to related vanadium(iv)-imido EPR data which are also isotropic.^42^

To probe the nature of the U–N–V linkage in 4 we performed DFT single point energy calculations on the 4′ and 4′′ spin-state formulations (Fig. S28–S31, Tables S1–S5, S7, ESI†). DFT computes 4′ to be 0.96 kJ mol^−1^ more stable than 4′′ which again suggests antiferromagnetic coupling. For 4′ the α-spin HOMO (268a) and HOMO−1 (267a) are 5f character, and the β-spin 267b orbital is the 2a_1g_ orbital of a bent metallocene (sd-hybrid). HOMOs−12, −20, and −21 reveal principally V–N π, π, and σ-bond interactions with weaker U–N π- and σ-components, respectively, and these interactions are also found in the NBO and NLMO analysis confirming that the V–N and U–N bonds are largely of imido and amido character, respectively. Inspection of 4′′ reveals a very similar bonding picture, except that after HOMO (269a) and HOMO−1 (268a) which are 5f-character the 2a_1g_ orbital is now found as HOMO−2 (267a) in the α- rather than β-spin manifold, and then the analogous V–N π, π, and σ-bond combinations are now HOMOs−13, −21, and −22. The computed bond order, charge, spin density, NBO, NLMO, and QTAIM data (Tables S1–S3 and S7, ESI†) are consistent with 4 being described as a U^IV^/V^IV^ complex where partial N-atom transfer from U to V has occurred.

To conclude, we have examined the reactivity of 1 towards a range of 3d-transition metal metallocenes. Although several metals (Cr, Mn, Fe, Ni) did not give tractable products, cobaltocene generated a uranium(v)-imido that results from one-electron reduction of uranium and nucleophilic attack of a cyclopentadienyl ligand by the nitride. In contrast, using decamethylcobaltocene resulted in two electron reduction of uranium and formation of a uranium(iv)-amido complex. The reaction of 1 with vanadocene resulted in a two electron redox couple resulting in U^IV^ and V^IV^ centres; since the nitride in 4 can be described as being formally of amido- and imido-type bonding character towards U and V, respectively, then 4 can be regarded as representing partial N-atom transfer from U to V. Nevertheless, there is clearly some electronic communication across the U–N–V linkage resulting in antiferromagnetic U–V exchange coupling. These complexes expand the still limited range of transition metal capped uranium-nitrides, and whilst demonstrating that constructing heterobimetallic actinide–nitride–metal linkages certainly benefits from starting with the nitride pre-installed at the actinide ion the resulting chemistry can still be complex and dictated by the nature of the transition metal fragment.

We thank the EPSRC (EP/K024000/1, EP/K024000/2, EP/M027015/1, EP/T011289/1), Royal Society (UF071260, UF110005, RG080285, RG110238, URF191320), ERC (239621, 612724, 851504), University of Nottingham, University of Manchester, Australian National University, and the University of Manchester Computational Shared Facility and the EPSRC UK EPR National Research Facility (EP/W014521/1 and EP/V035231/1) for supporting this work.

## Data availability

Data are available in the ESI,† from the CCDC, or from the authors on request. CCDC codes 2373740 (2) and 2373741 (4).

## Conflicts of interest

There are no conflicts to declare.

## Supplementary Material

CC-060-D4CC03846K-s001

CC-060-D4CC03846K-s002
