# Supplementary material for: Reactivity of a triamidoamine terminal uranium(vi)-nitride with 3d-transition metal metallocenes
Source: Chem Commun (Camb). 2024 Aug 15;60(73):9990–3. doi: 10.1039/d4cc03846k (PMC11342066; doi:10.1039/d4cc03846k)
Supplement: CC-060-D4CC03846K-s001 [file CC-060-D4CC03846K-s001.pdf]

Supplementary Information (SI) for ChemComm.
This journal is © The Royal Society of Chemistry 2024

– Supporting Information –

Reactivity of a Triamidoamine Terminal Uranium(VI)-Nitride with 3d-Transition Metal Metallocenes

John A. Seed,[a] Peter A. Cleaves,[a] Georgina R. Hatton,[a] David M. King,[b] Floriana Tuna,[c] Ashley J. Wooles,[a] Nicholas F. Chilton,[a,d]* Stephen T. Liddle[a]*

[a] *Department of Chemistry, The University of Manchester, Oxford Road, Manchester, M13 9PL, UK.*

[b] *School of Chemistry, The University of Nottingham, University Park, Nottingham, NG7 2RD, UK.*

[c] *Department of Chemistry and Photon Science Institute, The University of Manchester, Oxford Road, Manchester, M13 9PL, UK.*

[d] *Research School of Chemistry, The Australian National University, Sullivans Creek Road, Canberra, ACT, 2601, Australia.*

*Correspondence email: nicholas.chilton@anu.edu.au; steve.liddle@manchester.ac.uk

Experimental Details

General

All manipulations were carried out using Schlenk techniques, or an MBraun UniLab glovebox, under an atmosphere of dry dinitrogen. Solvents were dried by passage through activated alumina towers, degassed, and stored over potassium mirrors. Glassware was silylated prior to use. The compound $[(Tren^{TIPS})U^{VI}{\equiv}N]$ (**1**) was prepared according to published procedures,[1] and $[V^{II}(\eta^5\text{-}C_5H_5)_2]$, $[Cr^{II}(\eta^5\text{-}C_5H_5)_2]$, $[Mn^{II}(\eta^5\text{-}C_5H_5)_2]$, $[Fe^{II}(\eta^5\text{-}C_5H_5)_2]$, $[Co^{II}(\eta^5\text{-}C_5H_5)_2]$, $[Co^{II}(\eta^5\text{-}C_5Me_5)_2]$, and $[Ni^{II}(\eta^5\text{-}C_5H_5)_2]$ were purchased from Sigma Aldrich and sublimed prior to use.

Crystals were examined using either an Agilent Supernova equipped with an Eos CCD detector using Mo Kα radiation (λ = 0.71073 Å) or a GV1000 Atlas diffractometers, equipped with an AtlasS2 CCD

area detectors using Cu Kα radiation (λ = 1.54184 Å). Intensities were integrated from data recorded on narrow (1.0°) frames by *ω* rotation. Cell parameters were refined from the observed positions of all strong reflections in each data set. Gaussian grid face-indexed absorption corrections with a beam profile correction were applied. The structures were solved by direct methods and all non-hydrogen atoms were refined by full-matrix least-squares on all unique F^2 values with anisotropic displacement parameters with exceptions noted in the respective cif files. Except where noted, Hydrogen atoms were refined with constrained geometries and riding thermal parameters. CrysAlisPro[2] was used for control and integration, SHELXS[3,4] was used for structure solution, and SHELXL[5] and Olex2[6] were employed for structure refinement. ORTEP-3[7] and POV-Ray[8] were employed for molecular graphics. ^{1}H, ^{13}C{^{1}H}, and ^{29}Si{^{1}H} spectra were recorded on a Bruker 400 spectrometer operating at 400.1, 125.8, and 79.5 MHz, respectively; chemical shifts are quoted in ppm and are relative to tetramethylsilane (^{1}H, ^{13}C, ^{29}Si). FTIR spectra were recorded on a Bruker Alpha spectrometer with a Platinum-ATR module in the glovebox. UV/Vis/NIR spectra were recorded on a Perkin Elmer Lambda 750 spectrometer where data were collected in 1mm path length cuvettes and were run versus the appropriate reference solvent. Variable-temperature magnetic moment data were recorded in an applied direct current (DC) field of 0.1 or 0.5 Tesla on a Quantum Design MPMS3 superconducting quantum interference device magnetometer using recrystallised powdered samples. Samples were carefully checked for purity and data reproducibility between independently prepared batches. Samples were crushed with a mortar and pestle under an argon atmosphere and immobilised in an eicosane matrix within a borosilicate glass NMR tube to prevent sample reorientation during measurements. The tube was flame-sealed under dynamic vacuum ($1x10^{-3}$ mbar) to a length of approximately 3 cm and mounted in the centre of a drinking straw, with the straw fixed to the end of an MPMS 3 sample rod. Care was taken to ensure complete thermalisation of the sample before each data point was measured by employing delays at each temperature point and the sample was held at 1.8 K for 60 minutes before isothermal magnetisation measurements to account for slow thermal equilibration of the sample. Diamagnetic corrections were applied using tabulated Pascal constants

and measurements were corrected for the effect of the blank sample holders (flame sealed Wilmad NMR tube and straw) and eicosane matrix. Elemental microanalyses were carried out by Mr Martin Jennings at the Micro Analytical Laboratory, Department of Chemistry, University of Manchester.

Attempted Reactions of 1 with [M^{II}(η^5-C_5H_5)$_2$] (M = Cr, Mn, Fe, and Ni)

Cr: To a Schlenk flask was added a solid mixture of **1** (0.35 g, 0.40 mmol) and freshly sublimed [Cr^{II}(η^5-C_5H_5)$_2$] (0.073 g, 0.40 mmol). At –78 °C, toluene (20 mL) was added, and the solution was allowed to warm to room temperature before being stirred for 16 hours. Volatiles were removed *in vacuo* to afford a yellow-brown solid. Despite exhaustive attempts, an isolable product could not be obtained. ^{1}H NMR spectroscopic analysis of the solid (C_6D_6, 298 K) exhibited multiple resonances across the range δ +32 to –14 ppm.

Mn: To a Schlenk flask was added a solid mixture of **1** (0.35 g, 0.40 mmol) and freshly sublimed [Mn^{II}(η^5-C_5H_5)$_2$] (0.074 g, 0.40 mmol). At –78 °C, toluene (20 mL) was added, and the solution was allowed to warm to room temperature before being stirred for 16 hours. Volatiles were removed *in vacuo* to afford a brown solid. Despite exhaustive attempts, an isolable product could not be obtained. ^{1}H NMR spectroscopic analysis of the solid (C_6D_6, 298 K) exhibited multiple resonances across the range δ +28 to –6 ppm.

Fe: To a Schlenk flask was added a solid mixture of **1** (0.35 g, 0.40 mmol) and freshly sublimed [Fe^{II}(η^5-C_5H_5)$_2$] (0.074 g, 0.40 mmol). At –78 °C, toluene (20 mL) was added, and the solution was allowed to warm to room temperature before being stirred for 16 hours. Volatiles were removed *in vacuo* to afford a brown solid. Despite exhaustive attempts, an isolable product could not be obtained. ^{1}H NMR spectroscopic analysis of the solid (C_6D_6, 298 K) exhibited multiple resonances across the range δ +28 to –38 ppm.

Ni: To a Schlenk flask was added a solid mixture of **1** (0.35 g, 0.40 mmol) and freshly sublimed [Ni^{II}(η^5-C_5H_5)$_2$] (0.076 g, 0.40 mmol). At –78 °C, toluene (20 mL) was added, and the solution was allowed to warm to room temperature before being stirred for 16 hours. Volatiles were removed *in vacuo* to afford a brown solid. Despite exhaustive attempts, an isolable product could not be obtained. ^{1}H NMR spectroscopic analysis of the solid (C_6D_6, 298 K) exhibited multiple resonances across the range δ +28 to –38 ppm.

Preparation of [(Tren^{TIPS})U^V=N-(η^1:η^4-C_5H_5)Co^I(η^5-C_5H_5)] (2)

To a Schlenk flask was added a solid mixture of **1** (0.35 g, 0.40 mmol) and freshly sublimed [Co^{II}(η^5-C_5H_5)$_2$] (0.151 g, 0.80 mmol). At –78 °C, toluene (20 mL) was added, and the solution was allowed to warm to room temperature before being stirred for 16 hours. Volatiles were removed *in vacuo* to afford a dark red solid. Soluble residues were then extracted into hexanes (2 × 10 mL), and the resultant dark red solution was concentrated to approximately 5 mL and stored at 5 °C for 24 hours to afford **2** as red crystals, along with both crystalline **1** and [Co^{II}(η^5-C_5H_5)$_2$]. Despite exhaustive efforts, it was not possible to isolate **2** cleanly, with ^{1}H NMR spectroscopic analysis of the solid mixture revealing the three complexes present – **1**, **2**, and [Co^{II}(η^5-C_5H_5)$_2$] in varying ratios. Further analysis was conducted on this mixed component solid. ^{1}H NMR (C_6D_6, 298 K): δ 23.48 (6H, br, CH_2), 17.93 (2H, br, C_4H_4), 10.55 (2H, br, C_4H_4), 9.71 (5H, br, Cp*H*), 6.75 (6H, br, CH_2), –1.48 (1H, br, NC*H*), –3.94 (9H, br, ^{i}Pr-C*H*), –4.24 (54H, br, ^{i}Pr-CH_3) ppm. FTIR *v*/cm^{-1} (ATR): 2939 (m), 2887 (m), 2860 (m), 1461 (m), 1413 (w), 1380 (w), 1242 (m), 1184 (w), 1133 (m), 1107 (m), 1049 (m), 992 (m), 925 (m), 880 (m), 804 (m), 725 (s).

Reaction of 1 with [Co^{II}(η^5-C_5Me_5)$_2$] and Isolation of [(Tren^{TIPS})U^{IV}-NH_2] (3)

To a Schlenk flask was added a solid mixture of **1** (0.35 g, 0.40 mmol) and freshly sublimed [Co^{II}(η^5-C_5Me_5)$_2$] (0.132 g, 0.40 mmol). At –78 °C, toluene (20 mL) was added, and the solution was allowed to warm to room temperature before being stirred for 16 hours. Volatiles were removed *in vacuo* to

afford a turquoise-blue solid. Soluble residues were then extracted into hexanes (2 × 10 mL), and the resultant solution was concentrated to approximately 5 mL and stored at 5 °C for 24 hours to afford **3** as emerald-green crystals. Characterisation data matched that previously reported.[9]

Preparation of [(Tren^{TIPS})U^{IV}-N=V^{IV}(η^5-C_5H_5)$_2$] (4)

To a Schlenk flask was added a solid mixture of **1** (0.35 g, 0.40 mmol) and freshly sublimed [V^{II}(η^5-C_5H_5)$_2$] (0.072 g, 0.40 mmol). At –78 °C, toluene (20 mL) was added and the reaction mixture stirred for 10 minutes. The solution was then allowed to warm to room temperature slowly over one hour before being stirred for 72 hours. Volatiles were removed *in vacuo* to afford a dark red solid which was dried for two hours. Soluble residues were then extracted into hexanes (20 ml), and the resultant dark red solution was concentrated to approximately 5 mL and stored at −30 °C for 24 hours to afford **4** as red crystals. Yield: 0.34 g, 82%. Anal. Calc. for $C_{43}H_{85}N_5Si_3UV$: C, 50.26; H, 8.53; N, 6.51. Found: C, 50.32; H, 8.50; N, 6.31. ^{1}H NMR (C_6D_6, 298 K): δ 72.53 (10H, s, Cp*H*), 32.45 (6H, s, CH_2), 4.46 (6H, s, CH_2), –5.12 (9H, s, ^{i}Pr-C*H*), -6.56 (54H, s, ^{i}Pr-CH_3) ppm. ^{29}Si{^{1}H} (C_6D_6, 298 K): δ –76.2 ppm. FTIR *v*/cm^{-1} (ATR): 2940 (m), 2862 (m), 1460 (m), 1401 (w), 1381 (w), 1363 (w), 1259 (m), 1094 (m), 1037 (m), 1011 (m), 927 (m), 915 (m) 881 (m), 867 (m), 778 (s), 727 (s).

Quantum Chemical Calculations

Density Functional Theory Calculations

Complex **2** was geometry optimised without constraints and then a single point energy calculation was conducted on the optimised coordinates. Attempts to geometry optimise **4** resulted in the central N-atom of the U-N-V unit moving such that the U-N and V-N distances were both ~1.95 Å. Given the poor agreement of geometry optimised **4** to the experimentally observed crystal structure metrics, we used the crystal structure coordinates, froze the heavy atom positions, and geometry optimised the H-atom positions. Single point energy calculations were then performed on the resulting coordinates. Complex **2** was computed with a doublet (1 unpaired electron) formulation, and given the results of

the state-averaged complete active space self-consistent field (SA-CASSCF) calculations below we computed **4** with doublet (1 unpaired electron, **4'**) and quartet (3 unpaired electrons, **4''**) spin-states to probe any possible U-V antiferromagnetic coupling. The calculations were performed using the Amsterdam Density Functional (ADF) suite version 2017 with standard convergence criteria.[10,11] The DFT calculations employed Slater type orbital (STO) triple-ζ-plus polarisation all-electron basis sets (from the Dirac and ZORA/TZP database of the ADF suite). Scalar relativistic approaches (spin-orbit neglected) were used within the ZORA Hamiltonian[12-14] for the inclusion of relativistic effects and the local density approximation (LDA) with the correlation potential due to Vosko *et al* was used in all of the calculations.[15] Generalised gradient approximation (GGA) corrections were performed using the functionals of Becke and Perdew.[16,17] Natural Bond Order (NBO) and Natural Localised Molecular Orbital (NLMO) analyses were carried out with NBO 6.0.19.[18] The Quantum Theory of Atoms in Molecules analysis[19,20] was carried out within the ADF program. We quote Nalewajski-Mrozek bond orders since they reproduce expected bond multiplicities reliably in polar heavy atom structures whereas Mayer bond orders for polar bonds often do not conform with chemical intuition.[21] The ADF-GUI (ADFview) was used to prepare the three-dimensional plots of the electron density.

State-Averaged Complete Active Space Self-Consistent Field Calculations

SA-CASSCF calculations were performed with OpenMolcas v21.06.[22] Basis sets were exclusively of the ANO-RCC type, with VTZP quality for U, V and the bridging N atoms, VDZ quality for all other non-H atoms, and MB quality for all H atoms.[23-25] The DKH-2 relativistic Hamiltonian[26] and Cholesky decomposition of the two-electron integrals at a threshold of 10^{-8} were employed. The starting materials contain U^{VI} ($5f^0$) and V^{II} ($3d^3$), but the distribution of oxidation states in **4** is unknown *a priori*. Preliminary state-averaged complete active space self-consistent field (SA-CASSCF) calculations with and active space of 3 electrons in 12 orbitals (3d and 5f) examining high spin (S_{tot} = 3/2) and low spin (S_{tot} = 1/2) multiplicities suggest that the ground state is dominated by U^{IV} ($5f^2$) and V^{IV} ($3d^1$) configurations. Hence, it appears that $[V(Cp)_2]$ has doubly-reduced

[U(N)(Tren^{TIPS})] in this case. The 3d^1 configuration for V^{IV} defines five $S_V = 1/2$ roots in the basis of configuration state functions (CSFs) while the 5f^2 configuration for U^{IV} defines 21 $S_U = 1$ and 28 $S_U = 0$ roots; this would lead to 105 $S_{tot} = 3/2$ and 245 $S_{tot} = 1/2$ roots excluding charge transfer (CT) excitations. However, it is likely that this excitation space will be polluted with ligand to metal CT, metal to ligand CT or intervalence CT states. Hence, we performed SA-CASSCF calculation restricted to the subspace defined by product of the ^{2}D term of V^{IV} and the lowest-lying ^{3}H term of U^{IV}, comprising 55 $S_{tot} = 3/2$ and 55 $S_{tot} = 1/2$ roots, and then mix the resulting states with spin-orbit (SO) coupling. We note, however, that due to covalency and crystal field splitting that the assignment of these roots to the $^2D\otimes^3H$ space is only approximate. Indeed, the resulting states should include ^{m}K, ^{m}I, ^{m}H, ^{m}G and ^{m}F terms (where $m = 4$ for $S_{tot} = 3/2$ and $m = 2$ for $S_{tot} = 1/2$); however, upon projection of the wavefunction onto an angular momentum basis using irreducible spherical tensor methods,[27] only the ^{m}H, ^{m}G and ^{m}F terms were found. Hence, we then performed a CAS configuration interaction (CI) calculation for 105 $S_{tot} = 3/2$ and 175 $S_{tot} = 1/2$ roots (followed by SO coupling) using the optimised orbitals to attempt to capture the ^{3}H, ^{3}F, ^{3}P, ^{1}G and ^{1}D terms of U^{IV}. Projection of the resulting wavefunctions yielded more components of the desired spectrum (Table S4), however even still the maximal angular momentum of the ^{m}K terms ($L_{tot} = L_V + L_U = 2 + 5 = 7$) is not realised. This indicates that the significant covalency and crystal field splitting of the 3d and 5f orbitals quenches the total orbital angular momentum of the complex. None-the-less, we can project the SO states onto an angular momentum basis to inspect the low-lying spectrum of **4** (Table S4). The ground Kramers doublet is dominated by $S_{tot} = 1/2$ states, suggesting that the interaction between the V^{IV} and U^{IV} is antiferromagnetic. Clearly though, the states are very mixed in terms of angular momentum projections, which arises from non-trivial interplay between exchange coupling, crystal field splitting (covalency) and SO coupling effects. The ground Kramers doublet has easy axis anisotropy parallel to the U-V vector (Figure S33), and while some other doublets share this axis as one of their principal *g*-vectors, many do not and many show significant rhombicity. Hence, we have also reported the effective *g*-value along the U-V vector (g_{zz} in Table S5).

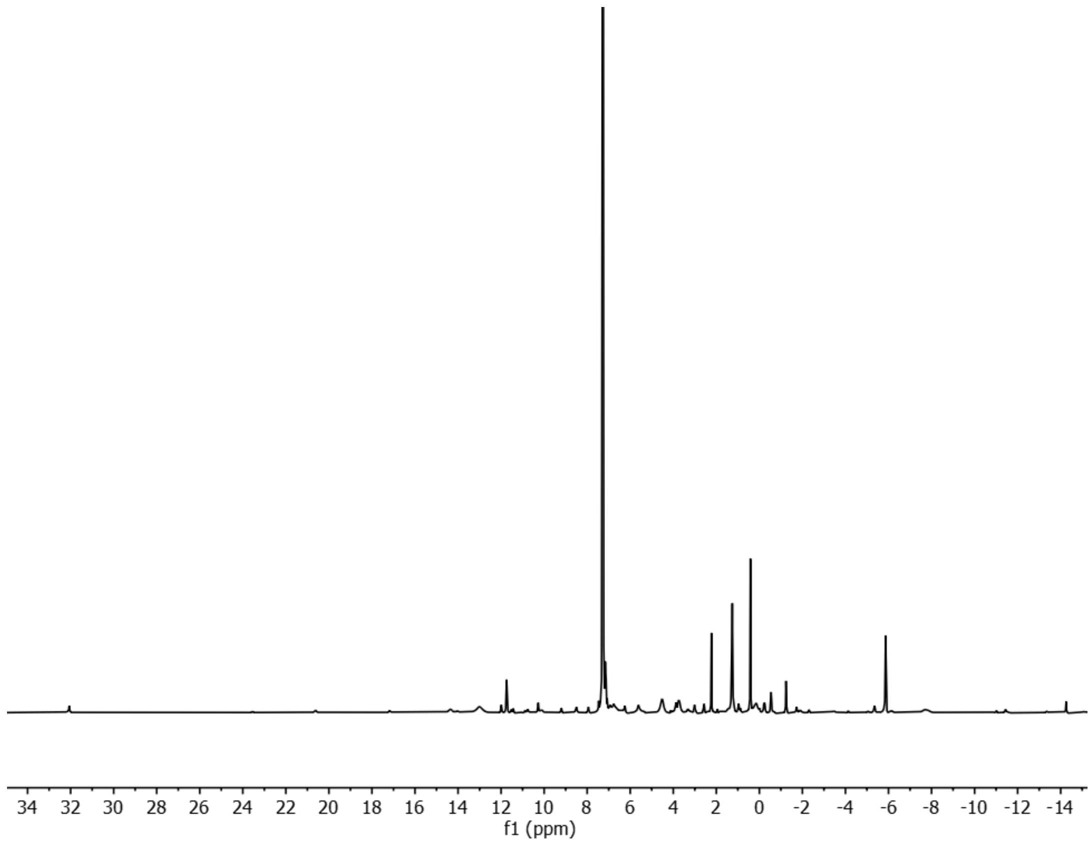

Figure S1. ^{1}H NMR (C_6D_6, 298 K) spectrum of the solid from the reaction of **1** with [Cr^{II}(η^5-C_5H_5)$_2$].

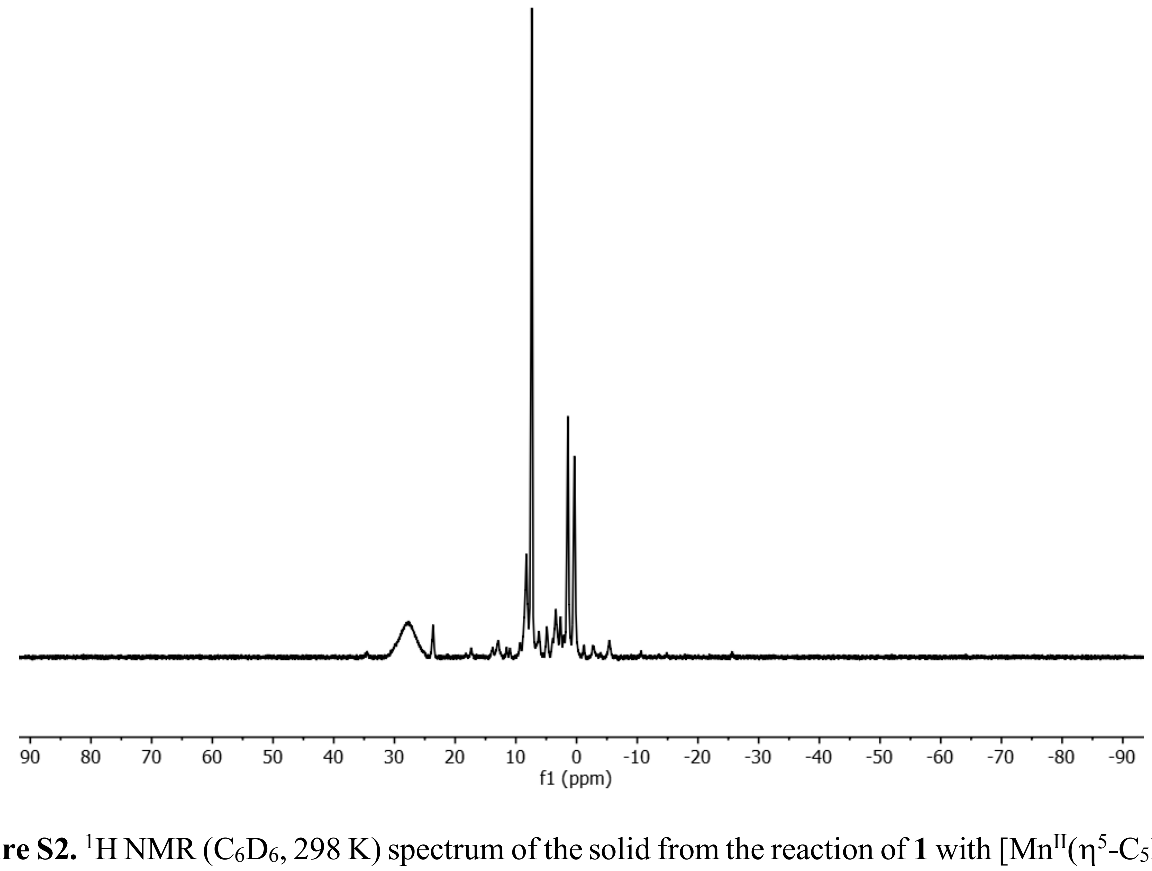

Figure S2. ^{1}H NMR (C_6D_6, 298 K) spectrum of the solid from the reaction of **1** with [Mn^{II}(η^5-C_5H_5)$_2$].

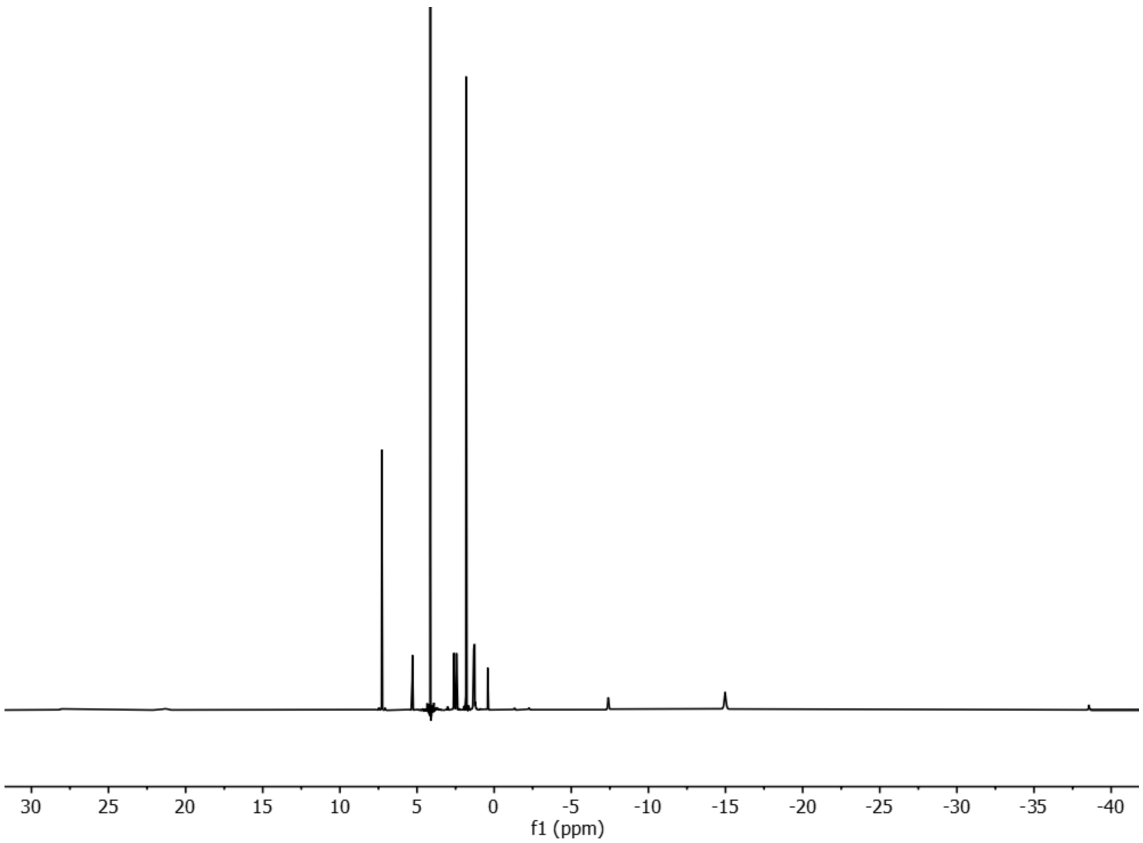

Figure S3. ^{1}H NMR (C_6D_6, 298 K) spectrum of the solid from the reaction of **1** with [$Fe^{II}(\eta^5\text{-}C_5H_5)_2$].

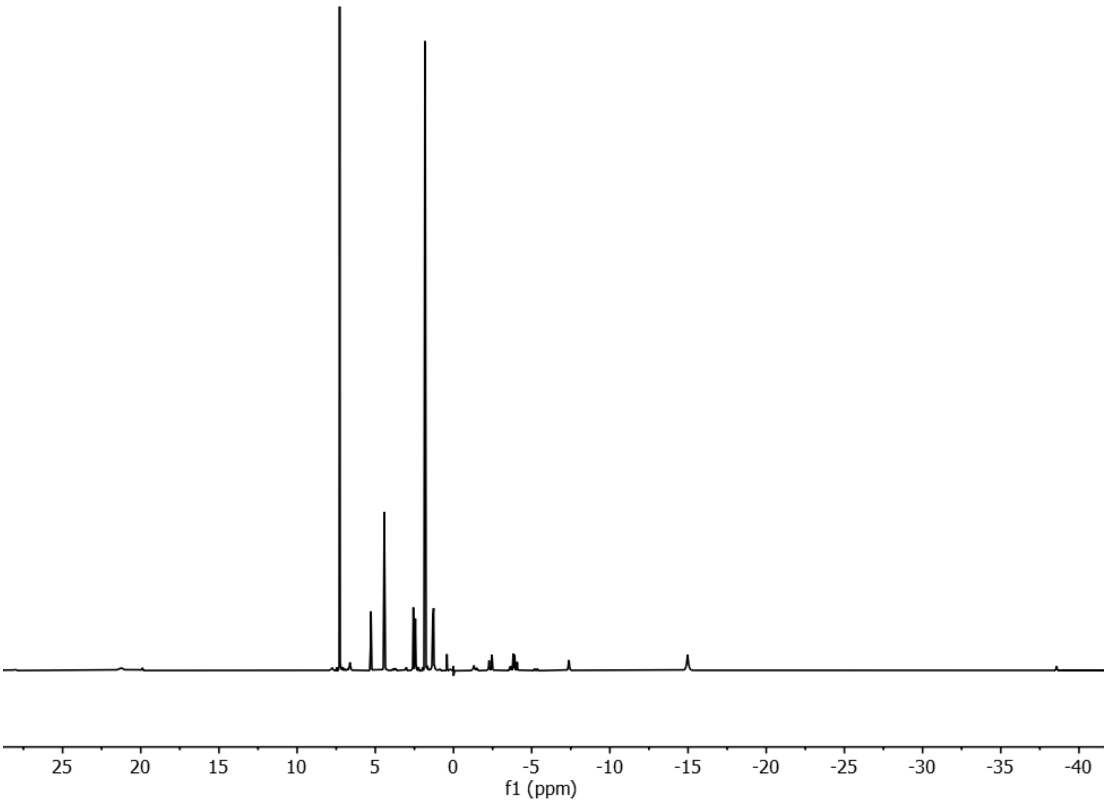

Figure S4. ^{1}H NMR (C_6D_6, 298 K) spectrum of the solid from the reaction of **1** with [$Ni^{II}(\eta^5\text{-}C_5H_5)_2$].

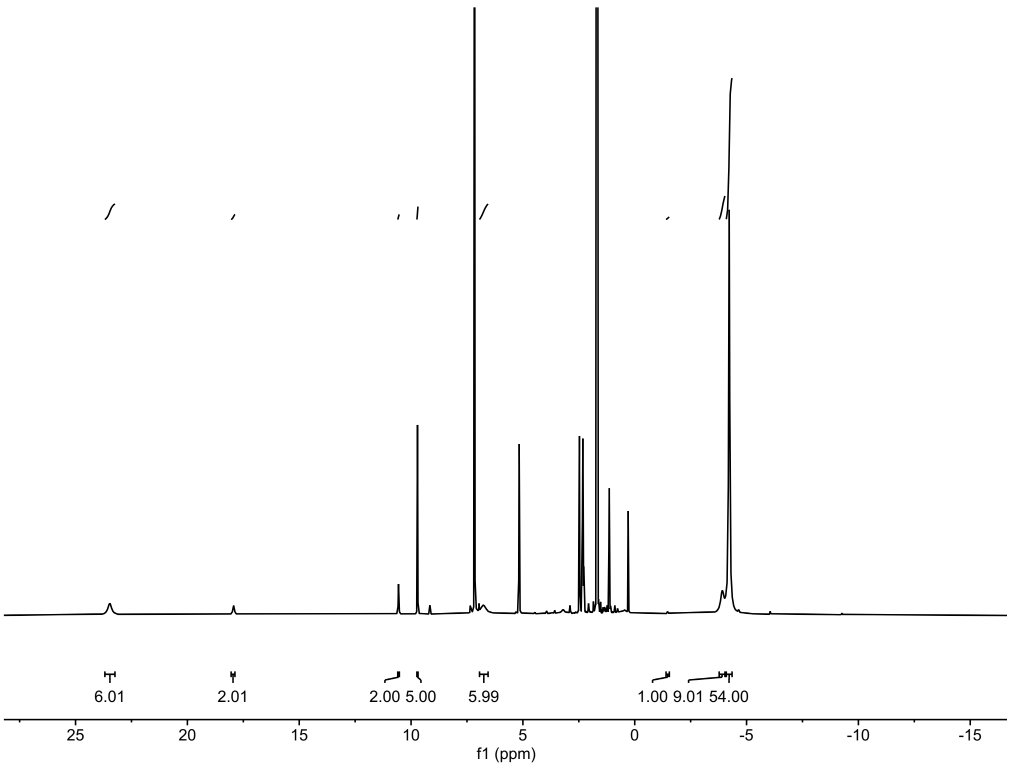

Figure S5. ^{1}H NMR (C_6D_6, 298 K) spectrum of **1**:**2**:[$Co^{II}(\eta^5-C_5H_5)_2$] (**A**) mixed component solid zoomed in between ~25 and –15 ppm to highlight the resonances attributed to **2**.

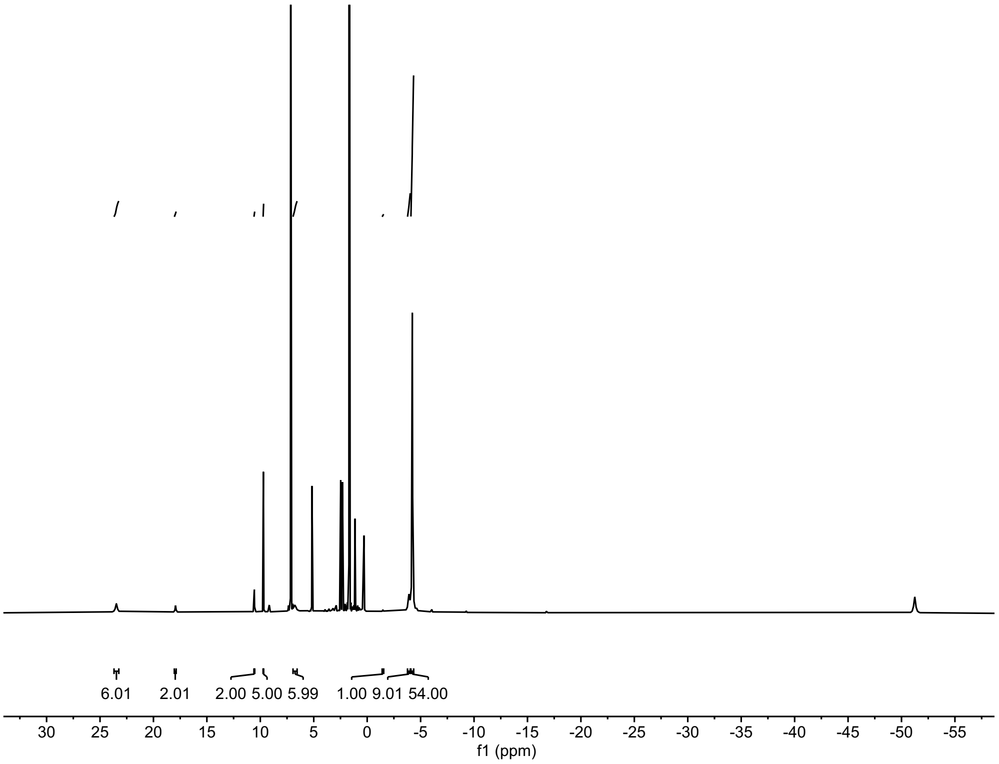

Figure S6. ^{1}H NMR (C_6D_6, 298 K) full spectrum of **1**:**2**:[$Co^{II}(\eta^5-C_5H_5)_2$] (**A**) mixed component solid; the resonance at 1.70 ppm is assigned as **1** (54H, d, ^{i}Pr-CH_3),[1] whilst the resonance at ~ –51 ppm is assigned as [$Co^{II}(\eta^5-C_5H_5)_2$].[28,29]

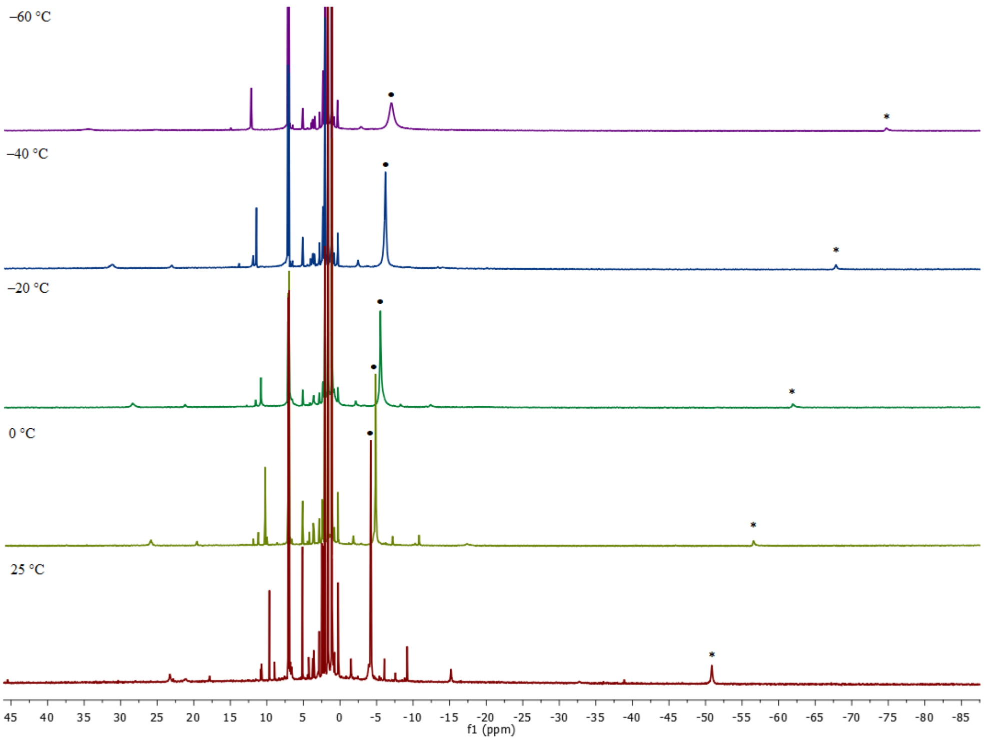

Figure S7. Variable-temperature NMR for a mixed component solution of **1**, **2** and [Co^{II}(η^5-C_5H_5)$_2$].

· signifies ^{i}Pr on **2** and * signifies [Co^{II}(η^5-C_5H_5)$_2$].

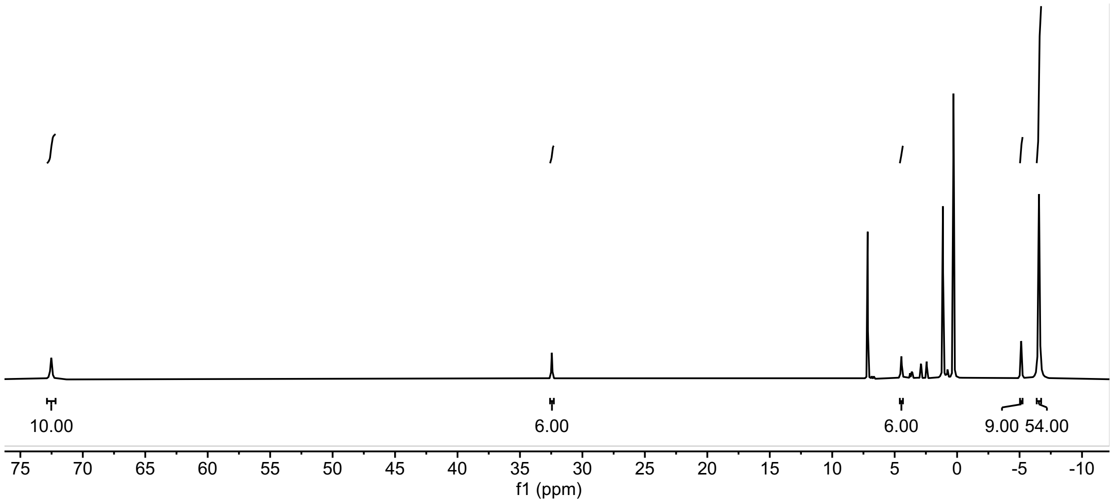

Figure S8. ^{1}H NMR (C_6D_6, 298 K) spectrum of **4**.

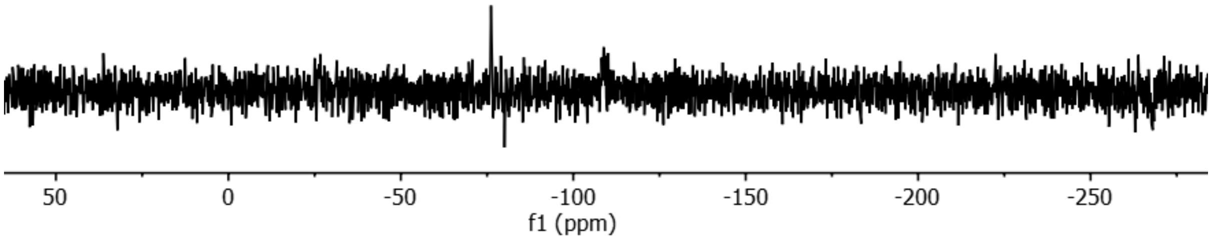

Figure S9. ^{29}Si{^{1}H} NMR (C_6D_6, 298 K) spectrum of **4**.

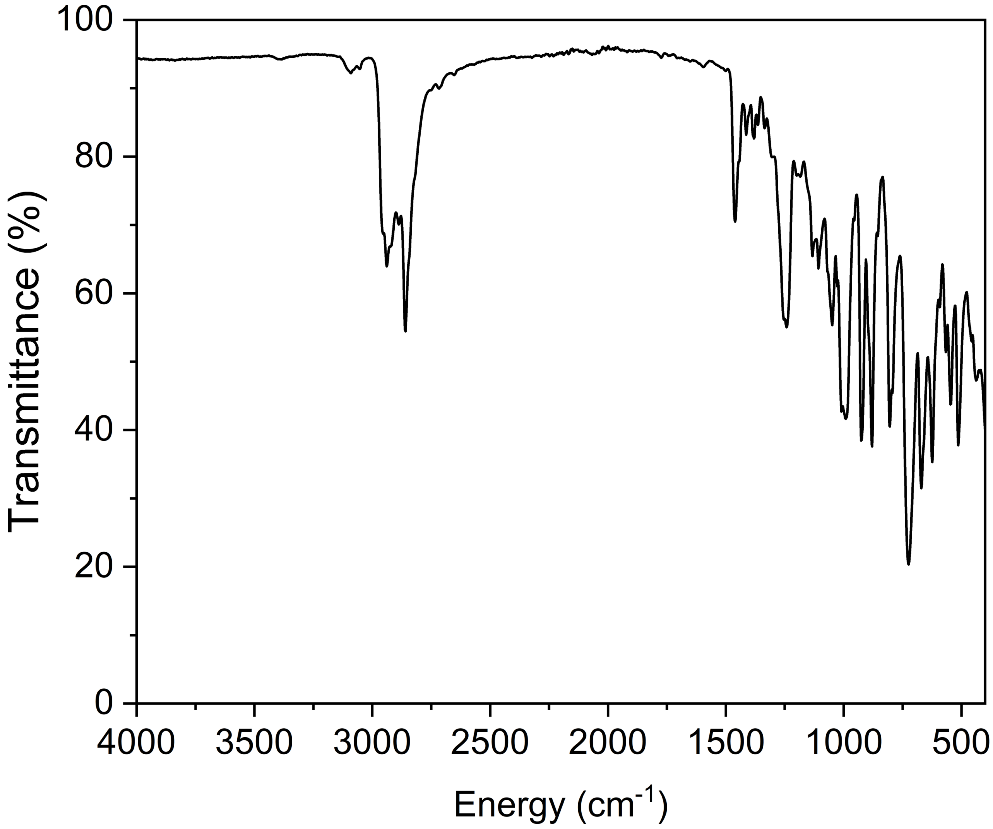

Figure S10. AT-IR spectrum of **1**:**2**:[Co^{II}(η^5-C_5H_5)$_2$] (**A**) mixed component solid.

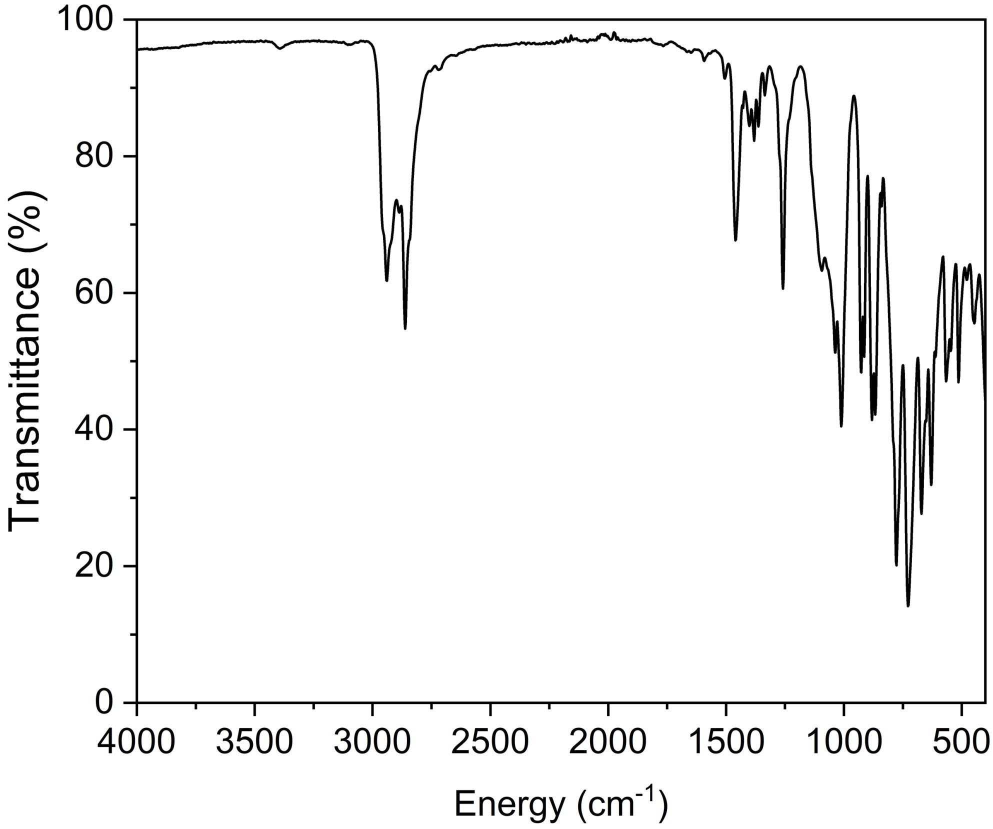

Figure S11. AT-IR spectrum of **4**.

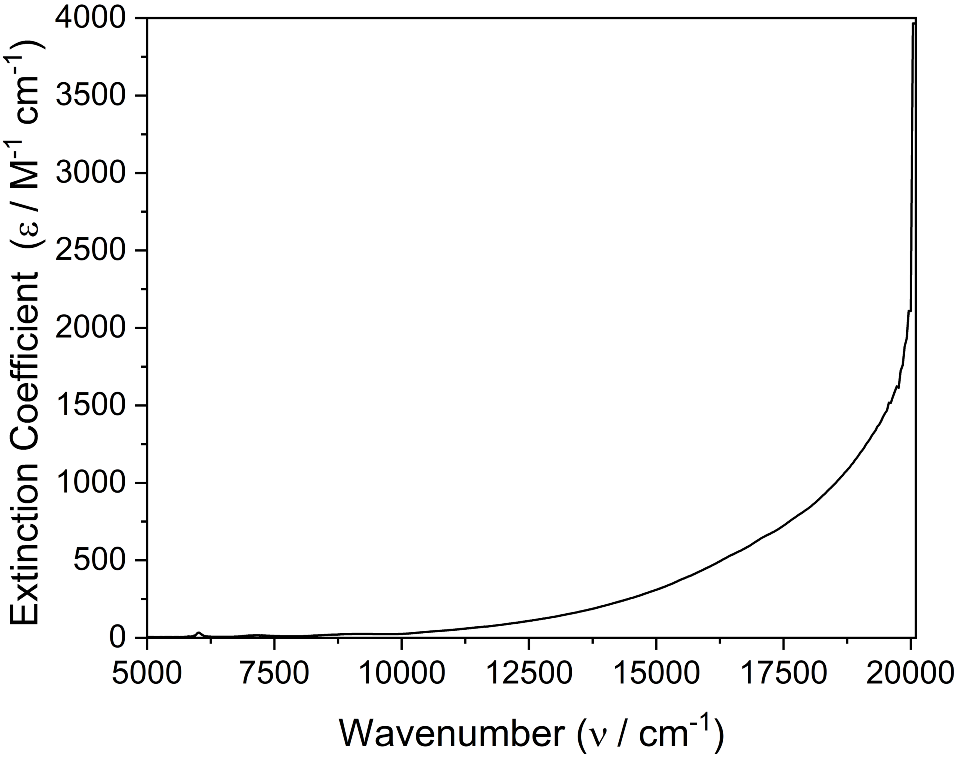

Figure S12. UV/Vis/NIR spectrum of **1**:**2**:[Co^{II}(η^5-C_5H_5)$_2$] (**A**) mixed component solid in toluene over the range 5000-20000 cm^{-1}.

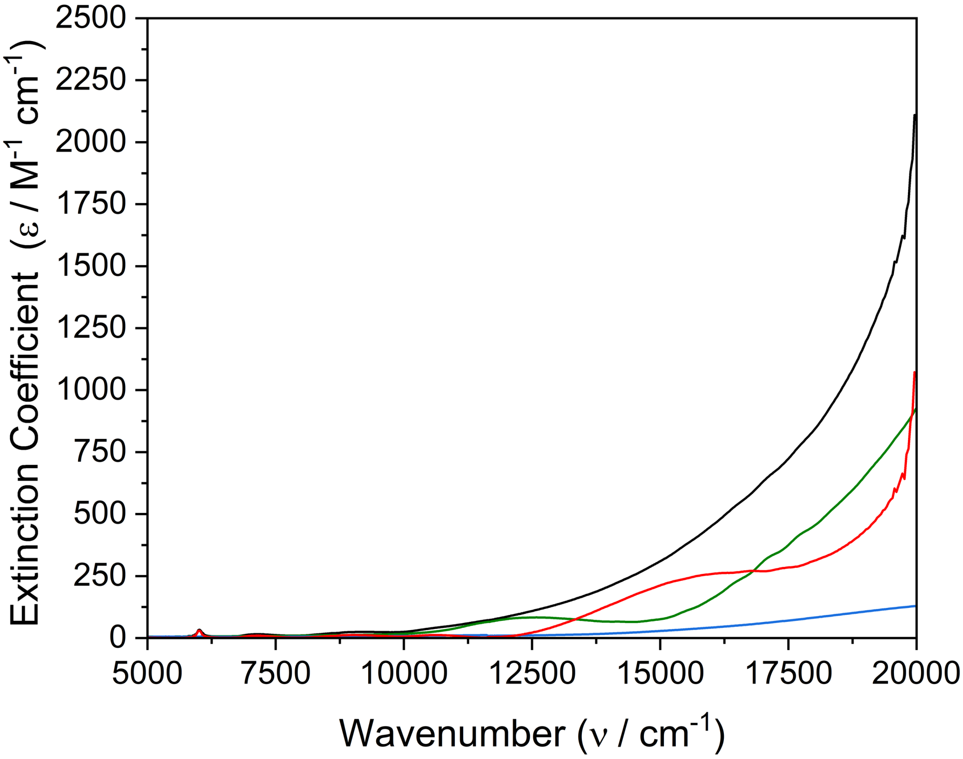

Figure S13. UV/Vis/NIR spectrum of **1** (green line), [Co^{II}(η^5-C_5H_5)$_2$] (blue line), **1**:**2**:[Co^{II}(η^5-C_5H_5)$_2$] (**A**) mixed component solid (black line), and **2** obtained by a subtraction method (red line) in toluene over the range 5000-20000 cm^{-1}.

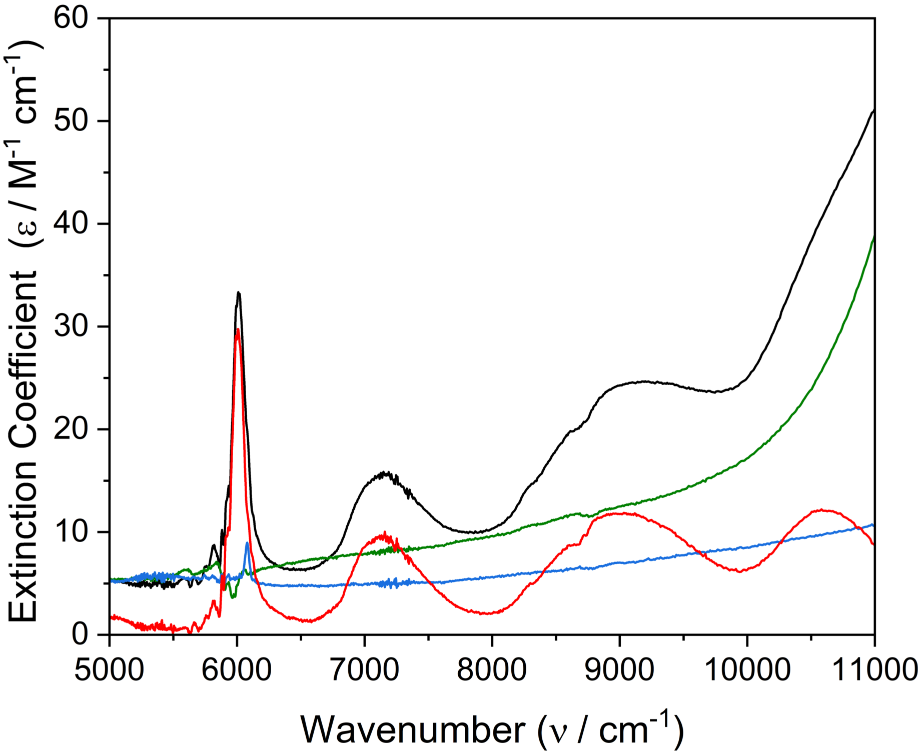

Figure S14. UV/Vis/NIR spectrum of **1** (green line), [Co^{II}(η^5-C_5H_5)$_2$] (blue line), **1**:**2**:[Co(Cp)$_2$] (**A**) mixed component solid (black line), and **2** obtained by a subtraction method (red line), in toluene zoomed in over the range 5000-11000 cm^{-1}.

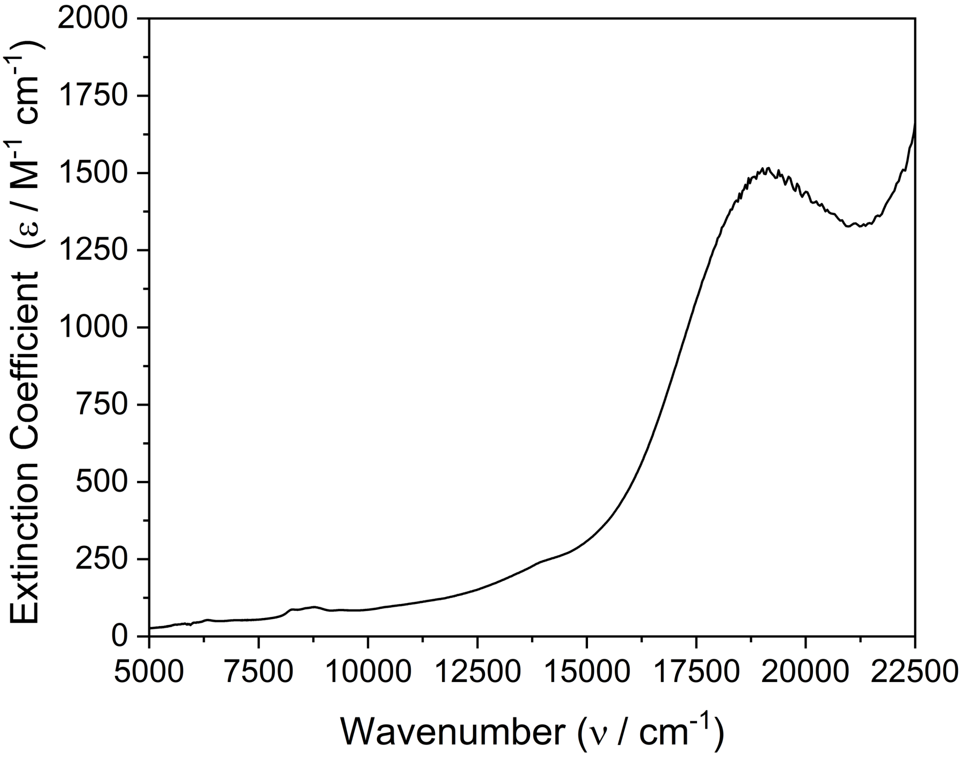

Figure S15. UV/Vis/NIR spectrum of **4** in toluene over the range 5000-22500 cm^{-1}.

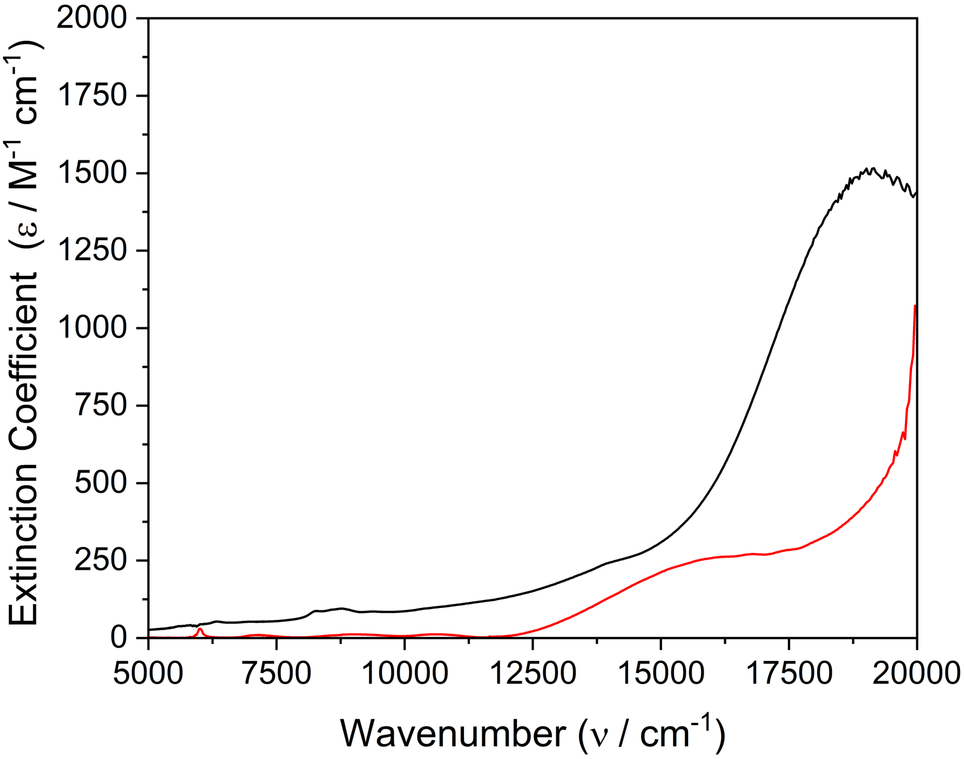

Figure S16. UV/Vis/NIR spectrum of **4** (black line), and **2** obtained by a subtraction method (red line), in toluene over the range 5000-20000 cm^{-1}.

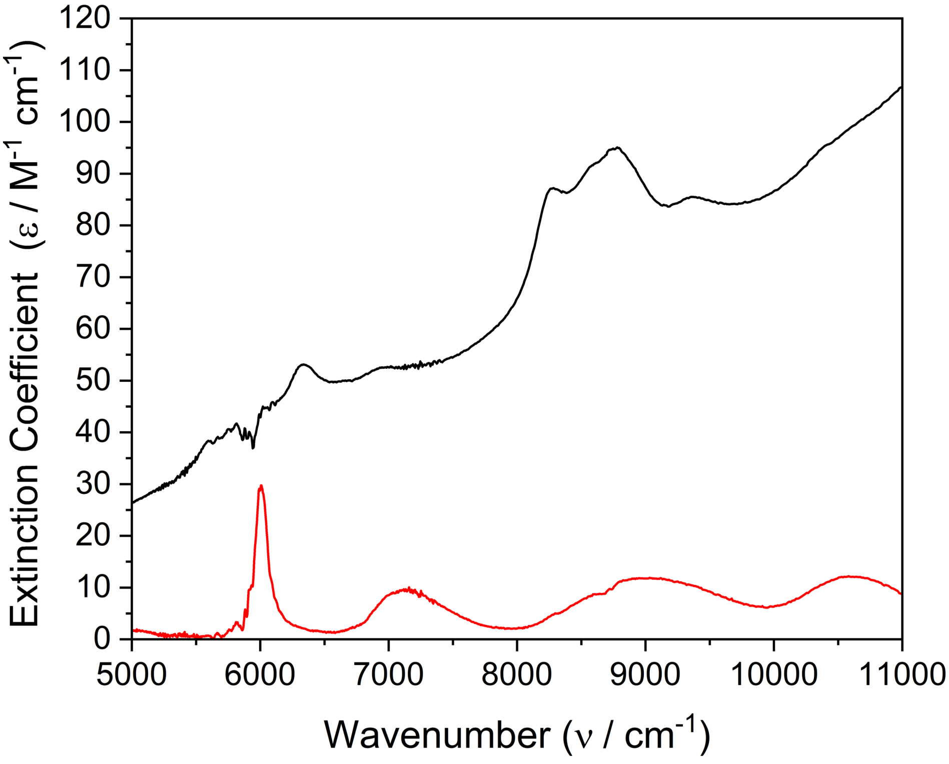

Figure S17. UV/Vis/NIR spectrum of **4** (black line), and **2** obtained by a subtraction method (red line), in toluene zoomed in over the range 5000-11000 cm^{-1}.

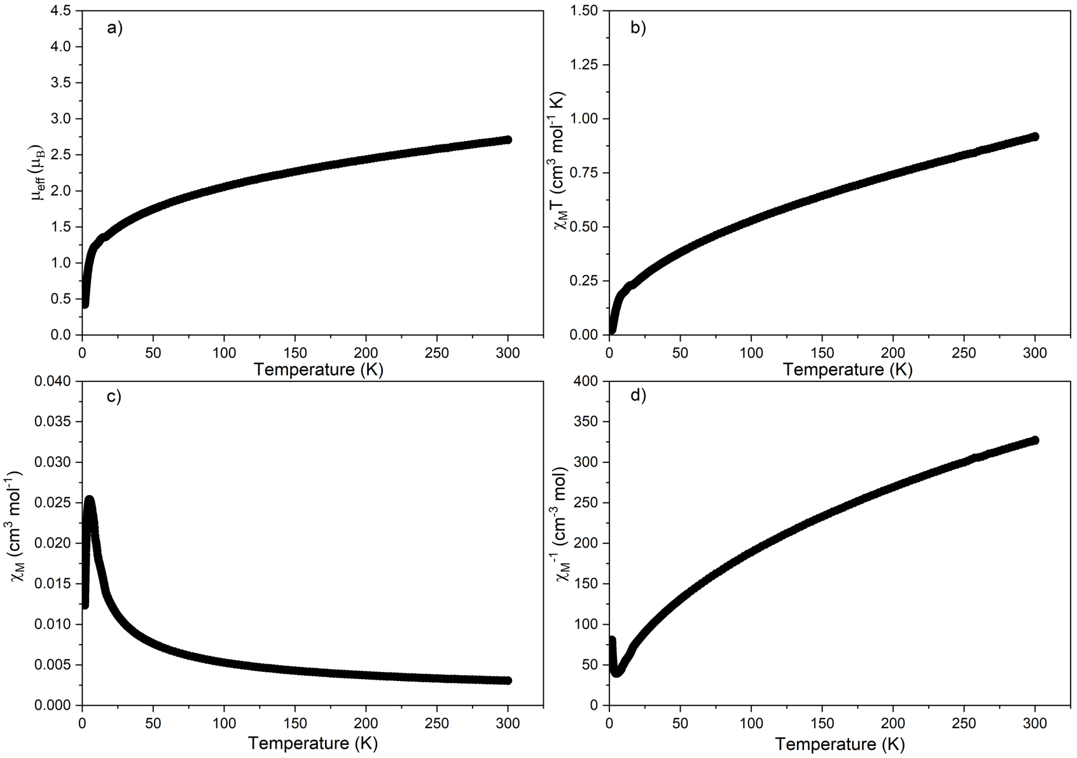

Figure S18. Variable-temperature SQUID magnetometry of **4** over the temperature range 1.8-300 K in an external 0.1 T field: a) μ_{eff} vs T; b) χT vs T; c) χ vs T; d) χ^{-1} vs T. Line is a guide to the eye only.

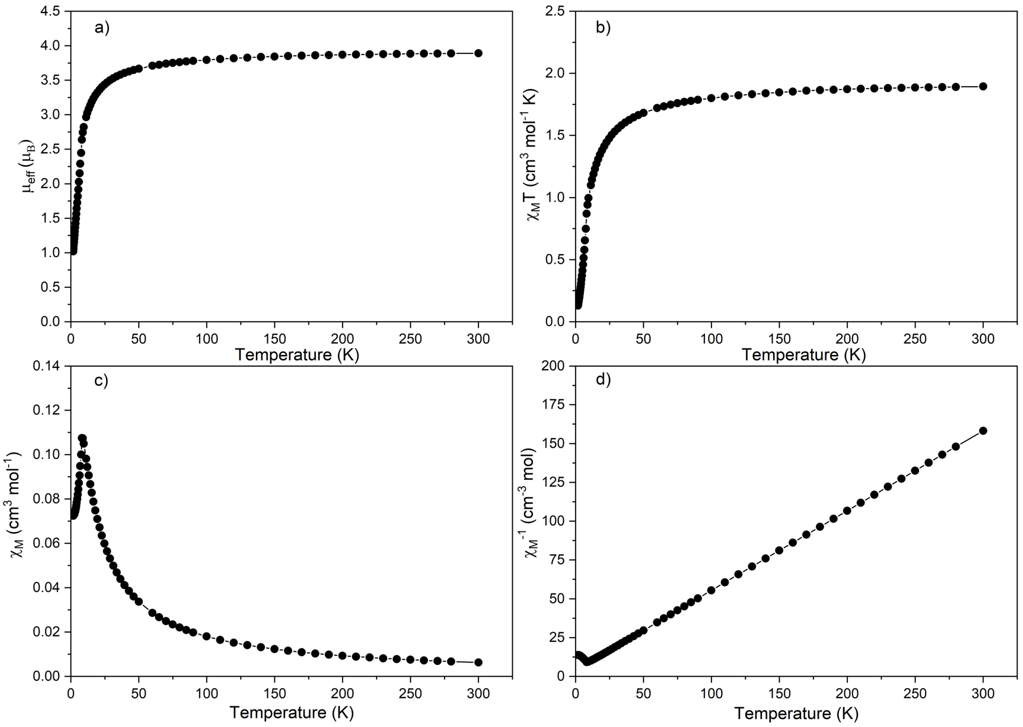

Figure S19. Variable-temperature SQUID magnetometry of $[V^{II}(\eta^5\text{-}C_5H_5)_2]$ over the temperature range 1.8-300 K in an external 0.5 T field: a) μ_{eff} vs T; b) χT vs T; c) χ vs T; d) χ^{-1} vs T. Line is a guide to the eye only.

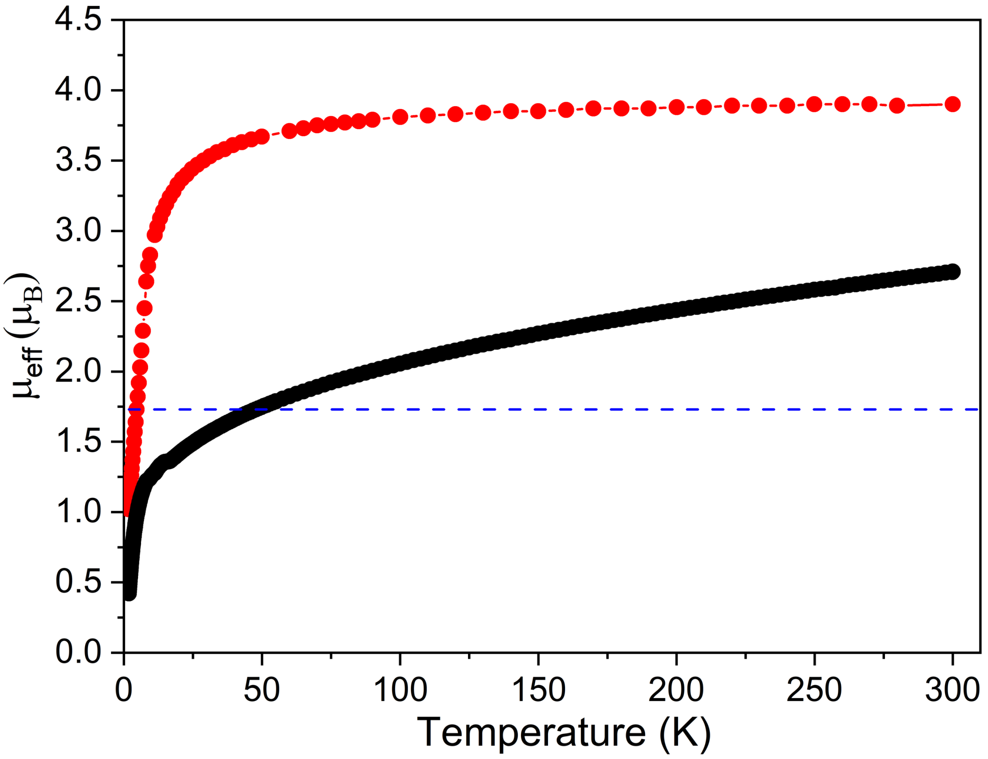

Figure S20. Temperature-dependent SQUID μ_{eff} (μ_B) vs Temperature (K) magnetometry data for **4** (black circles) and [V^{II}(η^5-C_5H_5)$_2$] (red circles) measured over the temperature range of 1.8 to 300 K. The black and red lines are a guide to the eye only, and the dashed line represents d^1 ($S = 1/2$, blue dashes) expected value for a $g = 2$ system.

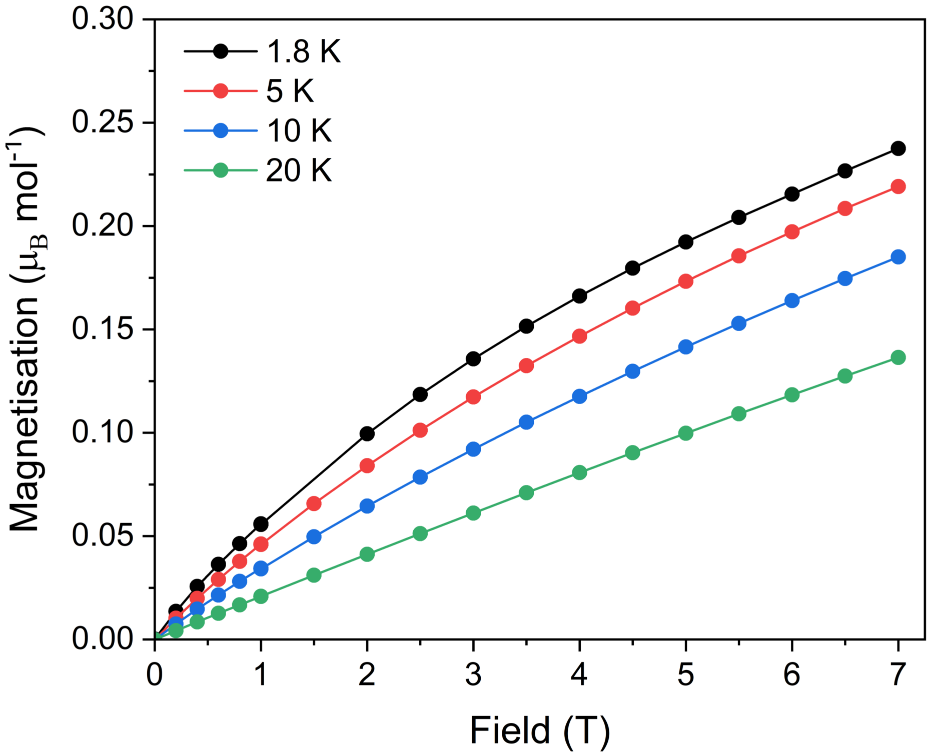

Figure S21. Magnetisation vs Field data for **4** at 1.8, 4, 10, and 20 K.

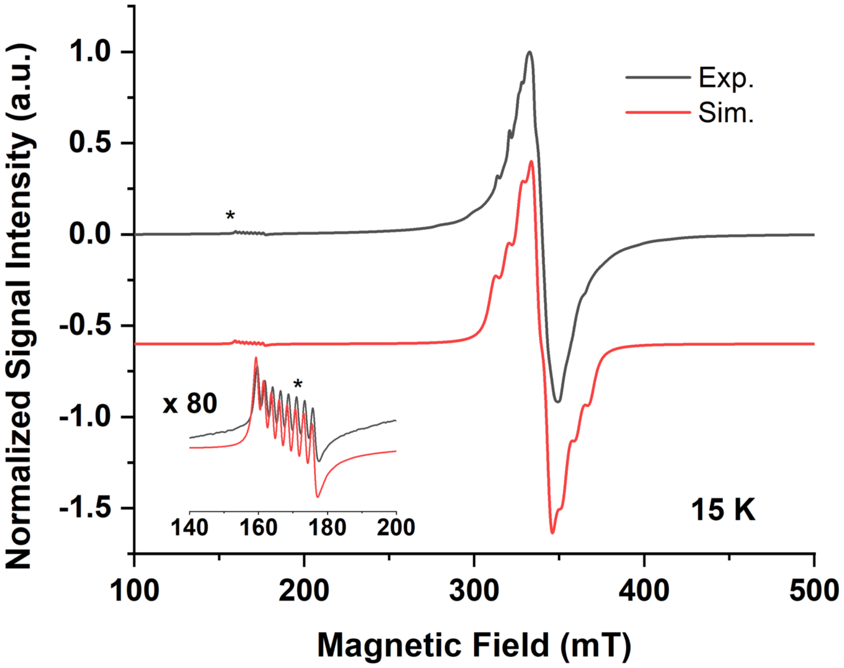

Figure S22. X-band (9.38 GHz) EPR spectrum of a powdered sample of **4** at 15 K (black) and its simulation (red) with $g = 1.971$, S = 1/2, $A_{x,y}(^{51}V) = 35$ MHz and $A_z(^{51}V) = 220$ MHz (red). The insert is the perpendicular component of a very small amount of $[V^{II}(\eta^5\text{-}C_5H_5)_2]$ impurity, corresponding to $g'_\perp = 3.996$ (or $g_\perp = 1.998$, considering S =3/2 and D>> hν) and $A_\perp(^{51}V) = 64$ MHz.

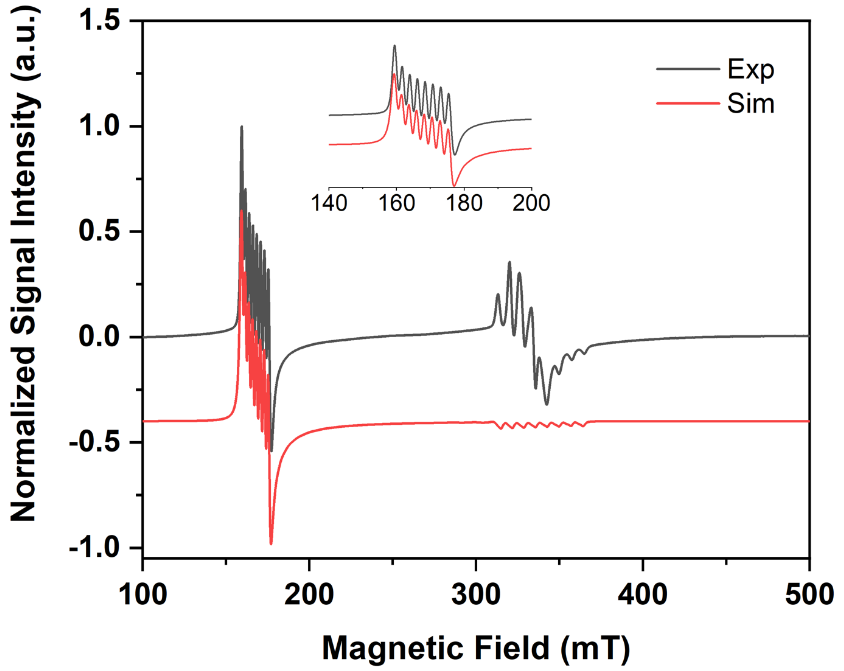

Figure S23. X-band (9.38 GHz) EPR spectrum of a powdered sample of pure $[V^{II}(\eta^5\text{-}C_5H_5)_2]$ at 10 K (black) and its simulation (red) with g = [1.998 1.998 1.970], A = [64, 64, 194] MHz, S = 3/2, |D| = 2.8 cm-1 >> hν. This is in excellent agreement with that previously reported for $[V^{II}(\eta^5\text{-}C_5H_5)_2]$.[30]

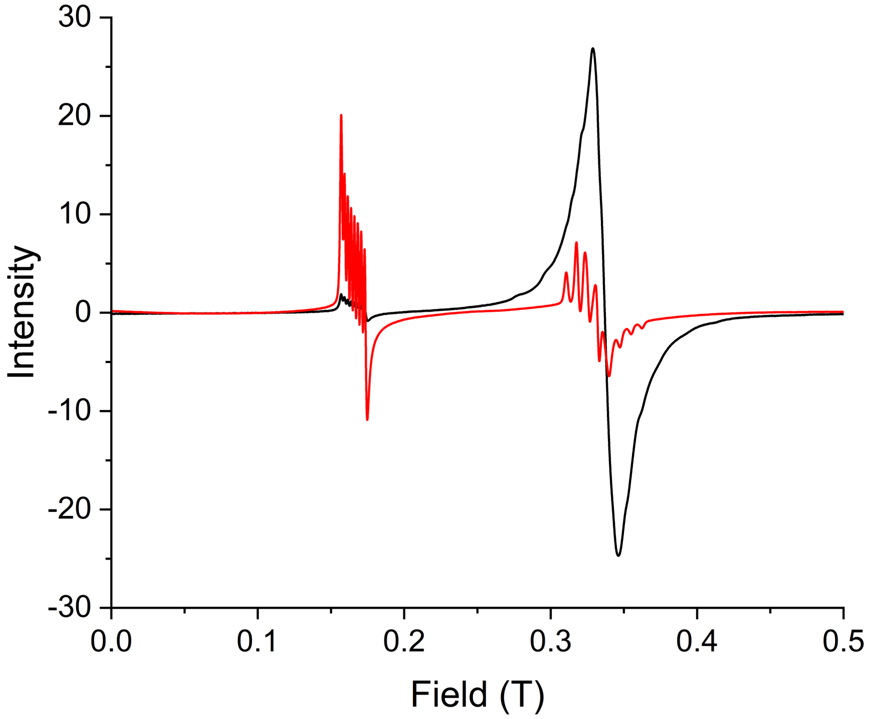

Figure S24. X-band (9.38 GHz) EPR spectrum of a powdered sample of **4** at 7 K (black line) and a powdered sample of pure [$V^{II}(\eta^5\text{-}C_5H_5)_2$] at 10 K (red line).

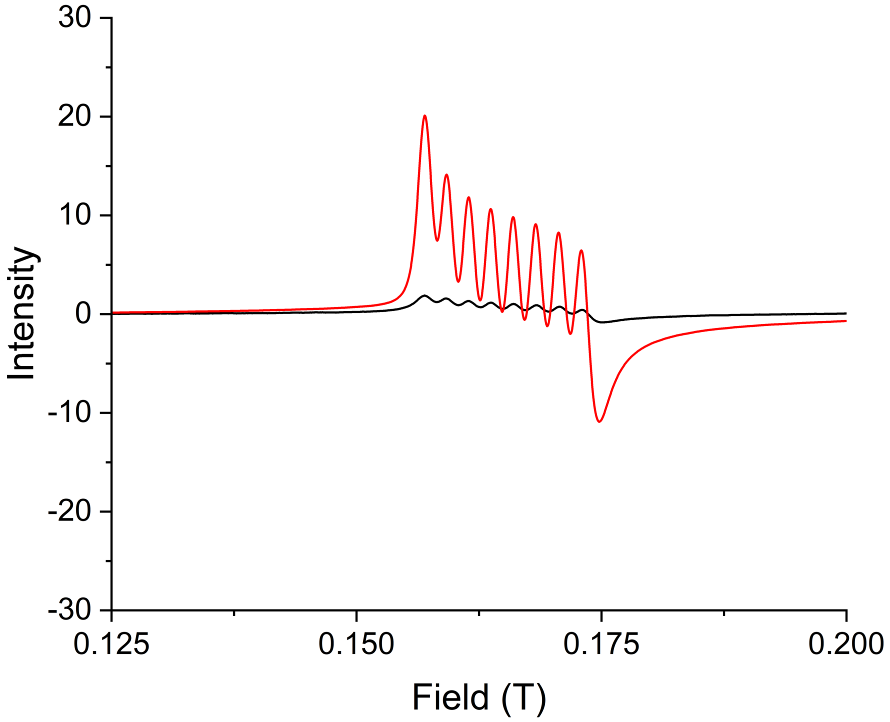

Figure S25. X-band (9.38 GHz) EPR spectrum of a powdered sample of **4** at 7 K (black line) and a powdered sample of pure [$V^{II}(\eta^5\text{-}C_5H_5)_2$] at 10 K (red line), zoomed in between 0.125 and 0.200 T.

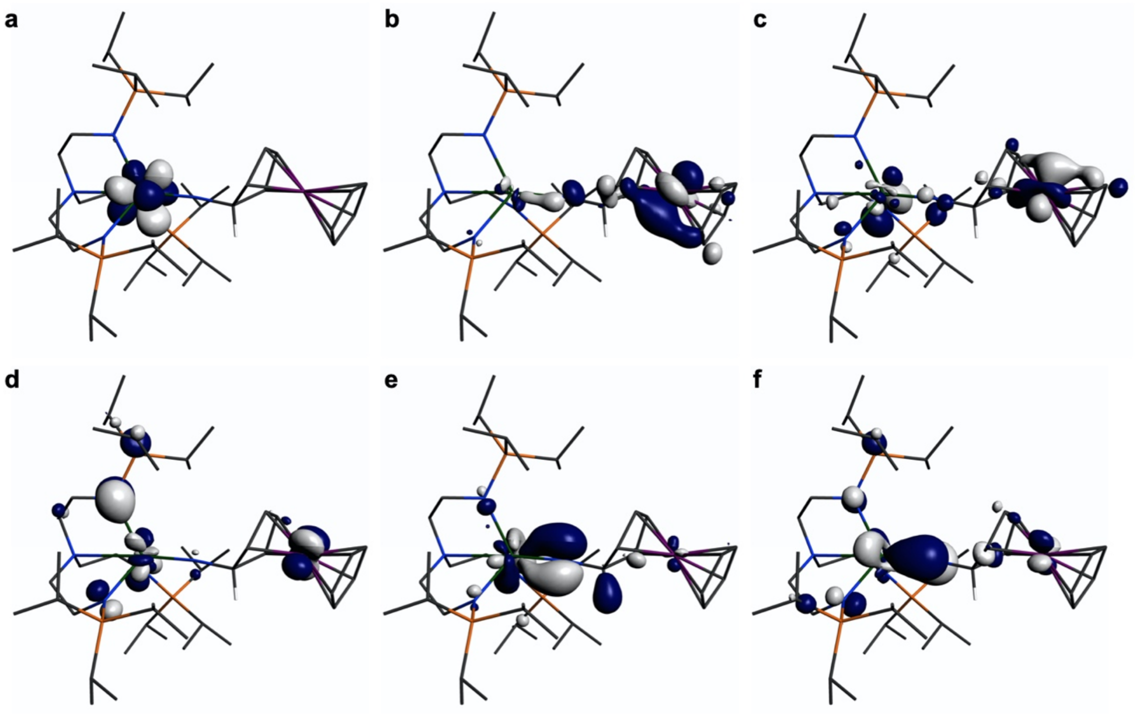

Figure S26. Selected α-spin frontier molecular orbitals of **2**. (a) HOMO (270a, −2.915 eV). (b) HOMO−1 (269a, −4.236 eV). (c) HOMO−4 (266a, −4.724 eV). (d) HOMO−5 (265a, −4.821 eV). (e) HOMO−8 (262a, −5.126 eV). (f) HOMO−9 (261a, −5.254 eV).

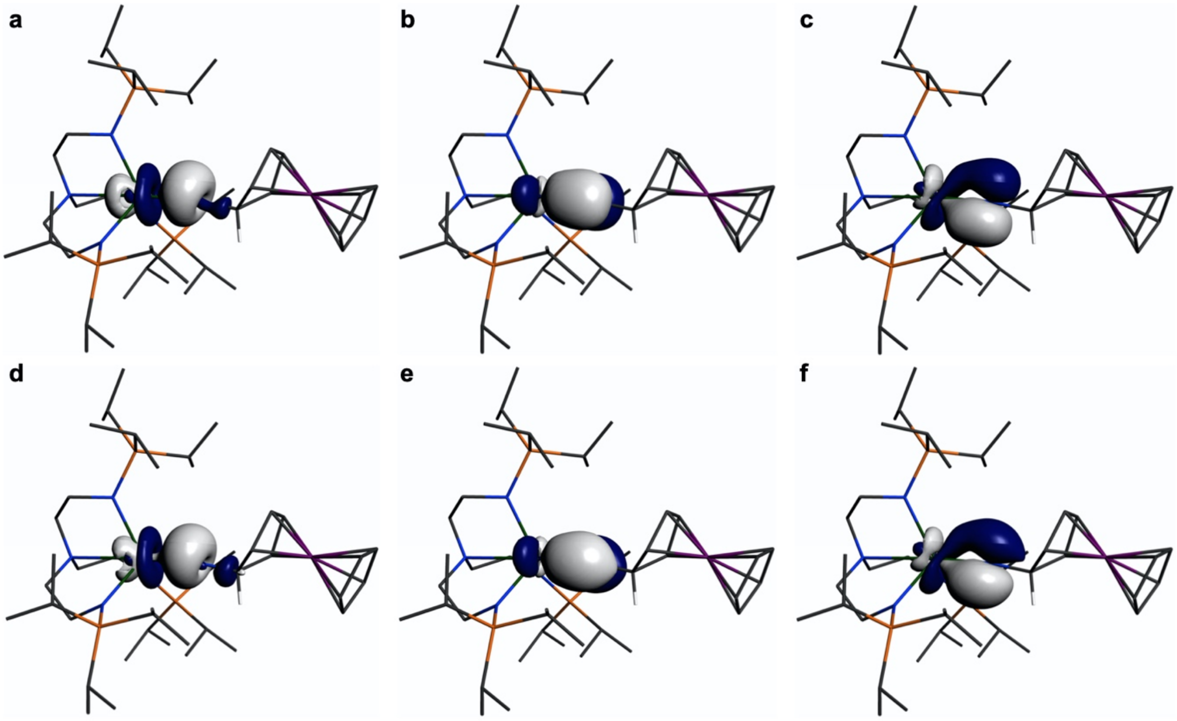

Figure S27. Selected NBO and NLMO representations of **2**. (a) NBO UN σ-bond. (b) NBO UN π-bond. (c) NBO UN π-bond. (d) NLMO UN σ-bond. (e) NLMO UN π-bond. (f) NLMO UN -π-bond.

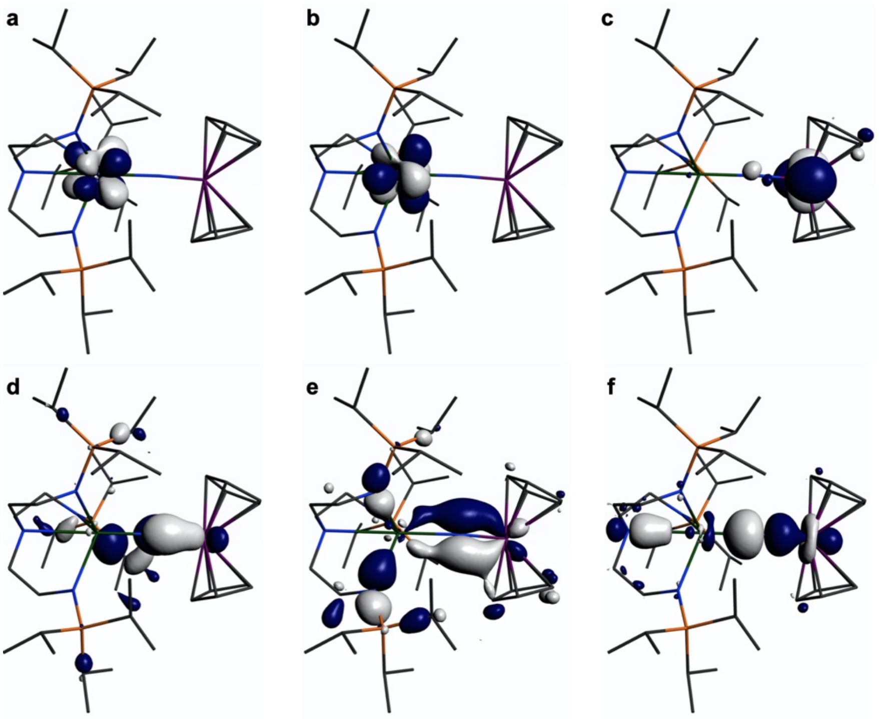

Figure S28. Selected α- and β-spin frontier molecular orbitals of S = 1/2 **4'**. (a) HOMO (268a, –2.412 eV). (b) HOMO–1 (267a, –2.420 eV). (c) HOMO–1 (267b, –3.761 eV). (d) HOMO–12 (256a, –5.849 eV). (e) HOMO–20 (248a, –6.581 eV). (f) HOMO–21 (247a, –6.889 eV).

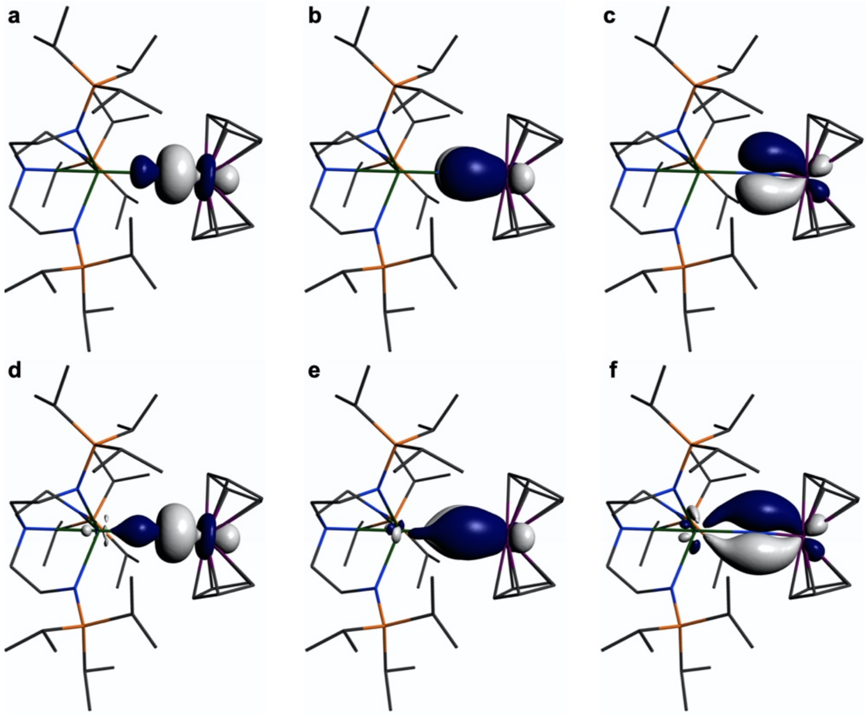

Figure S29. Selected NBO and NLMO representations of S = 1/2 **4'**. (a) NBO VN σ-bond. (b) NBO VN π-bond. (c) NBO VN π-bond. (d) NLMO VN σ-bond. (e) NLMO VN π-bond. (f) NLMO VN -π-bond.

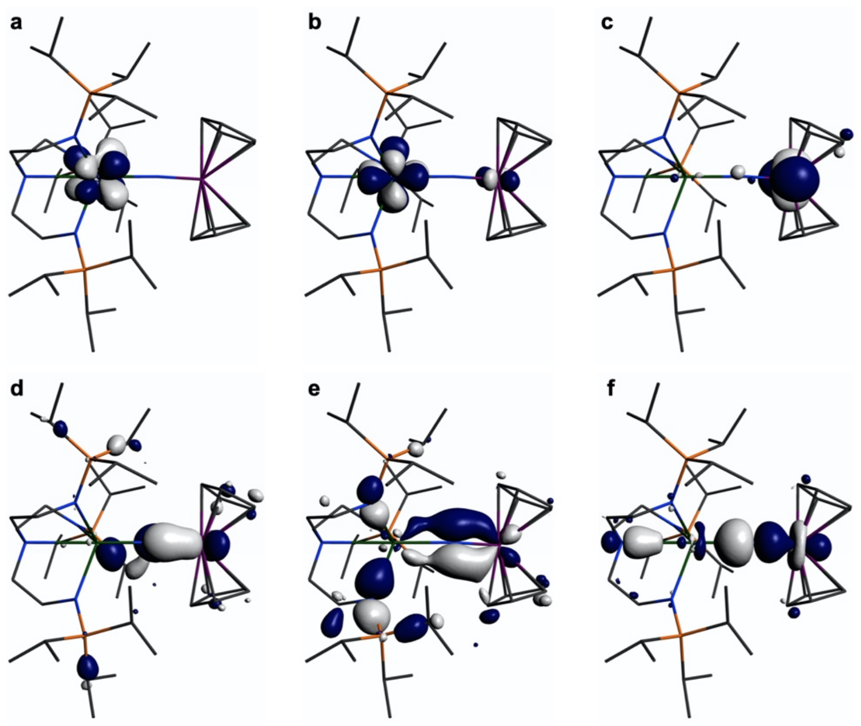

Figure S30. Selected α-spin frontier molecular orbitals of S = 3/2 **4''**. (a) HOMO (269a, −2.528 eV). (b) HOMO−1 (268a, −2.570 eV). (c) HOMO−2 (267a, −3.683 eV). (d) HOMO−13 (256a, −5.872 eV). (e) HOMO−21 (248a, −6.597 eV). (f) HOMO−22 (247a, −6.932 eV).

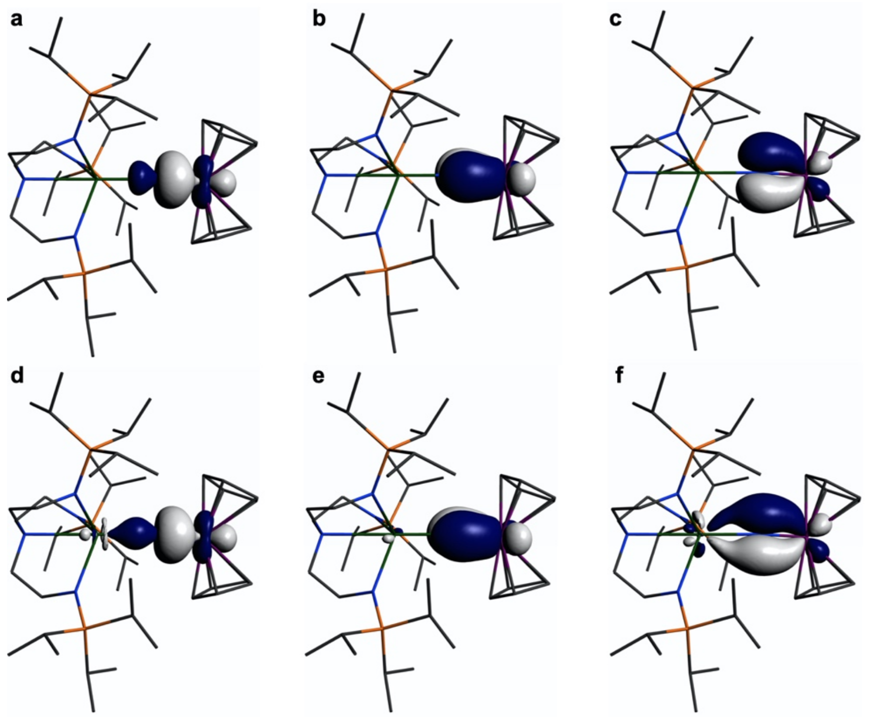

Figure S31. Selected NBO and NLMO representations of S = 3/2 **4''**. (a) NBO VN σ-bond. (b) NBO VN π-bond. (c) NBO VN π-bond. (d) NLMO VN σ-bond. (e) NLMO VN π-bond. (f) NLMO VN -π-bond.

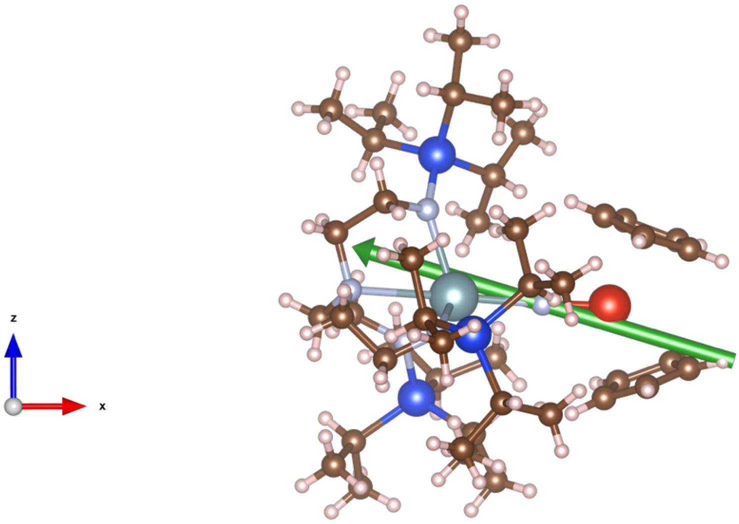

Figure S32. Illustration of the easy axis anisotropy of the Kramers doublet of **4**.

Tables

Table S1. Selected computed bond orders, charges, and spin-densitites, for 2, 4', and 4''

Cmpd	Bond Order[a]		Charge[b]			Spin density[c]		
	UN	Co/VN	U	N	Co/V	U	N	Co/V
**2**	2.73	-	3.02	−1.19	0.22	1.30	−0.13	−0.01
**4'**	1.40	2.42	2.52	−1.04	0.80	2.33	0.15	−1.26
**4''**	1.50	2.15	2.53	−1.05	0.81	2.33	−0.29	1.32

[a] Nalewajski-Mrozek bond order. [b] Multipole Derived Charge (quadrupole) atomic charge. [c] Multipole Derived Charge (monopole) atomic spin-density.

Table S2. Selected NLMO compositions of 2, 4', and 4''

Cmpd	Bond	U%	N%	Co/V%	U 7s/7p/6d/5f	N 2s/2p	Co/V 4s/4p/3d
**2**	σ	9	89	-	3/2/42/53	36/64	-
	π	19	78	-	0/0/20/80	0/100	-
	π	21	77	-	0/0/17/83	0/100	-
**4'**	σ	2	65	33	7/3/42/48	26/74	2/0/98
	π	4	59	37	0/0/30/70	0/100	0/0/100
	π	5	60	35	0/0/25/75	0/100	0/0/100
**4''**	σ	3	65	32	3/2/28/67	24/76	2/0/98
	π	3	56	41	1/1/50/48	0/100	0/0/100
	π	9	59	32	0/0/21/79	0/100	0/0/100

Table S3. Selected QTAIM properties of 2, 4', and 4''

Cmpd	Bond	ρ	$\nabla^2\rho$	H	ε	Bond	ρ	$\nabla^2\rho$	H	ε
**2**	U-N	0.18	0.43	−0.13	0.03	Co-N	-	-	-	-
**4'**	U-N	0.09	0.22	−0.04	0.07	V-N	0.22	0.64	−0.17	0.01
**4''**	U-N	0.10	0.24	−0.05	0.12	V-N	0.22	0.64	−0.17	0.03

Table S4. Angular momentum content for the ab initio wavefunction of 4 calculated with CASCI-SO and projected onto an angular momentum basis. Energies rounded to the nearest cm^{-1}, wavefunction contributions rounded to the nearest percent and only components > 10% shown.

**Term**	**Quantity**	
	**m = 2**	**m = 4**
^{m}K	0.92	0.27
^{m}I	1.82	1.18
^{m}H	3.84	2.13
^{m}G	4.66	3.28
^{m}F	4.46	2.48
^{m}D	3.49	2.26
^{m}P	1.75	1.45
^{m}S	0.23	0.45

Table S5. Low-lying spectrum of Kramers doublets in 4 calculated with CASCI-SO and projected onto an angular momentum basis. Energies rounded to the nearest cm^{-1}, wavefunction contributions rounded to the nearest percent and only components > 10% shown. Subsequent excited states > 1800 cm^{-1}. Effective g-values are calculated for each Kramers doublet using the pseudo-spin S = 1/2 convention, hence the reported eigenvalues have their own reference frame, and show no symmetries. The projected effective g-value along the U-V vector is reported as g_{zz}.

**Energy (cm^{-1})**	g_1	g_2	g_3	g_{zz}	**Wavefunction**
0	6.80	0.35	0.24	6.48	15%|$^2F_{5/2}$>+13%|$^2I_{11/2}$>+12%|$^2I_{13/2}$>+11%|$^2K_{13/2}$>
56	4.08	3.25	2.12	2.16	19%|$^2I_{11/2}$>+16%|$^2G_{7/2}$>+11%|$^4I_{11/2}$>
374	2.74	1.46	1.23	2.88	25%|$^4I_{9/2}$>+15%|$^4I_{11/2}$>+12%|$^4K_{11/2}$>+11%|$^4F_{3/2}$>+11%|$^4H_{7/2}$>+10%|$^4G_{5/2}$>
811	3.89	0.84	0.69	3.90	14%|$^2I_{11/2}$>+11%|$^4I_{11/2}$>
970	2.18	0.44	0.26	0.06	17%|$^4I_{9/2}$>+10%|$^4I_{11/2}$>
1357	4.02	1.00	0.55	3.74	-
1547	4.33	1.57	0.39	3.53	10%|$^2H_{9/2}$>+10%|$^2I_{11/2}$>
1619	1.43	0.74	0.36	1.31	19%|$^4I_{9/2}$>+12%|$^4G_{5/2}$>+12%|$^4H_{7/2}$>

Table S6. Final coordinates and single point energy for 2

1.C	-0.885623	-1.735471	-4.943353
2.C	-0.534138	-4.250808	-4.831578
3.C	-1.221262	-3.031205	-4.180173
4.C	-2.956084	3.178270	-3.765006
5.C	-2.241784	5.538631	-3.206851
6.C	0.170487	1.554952	-3.279874
7.C	1.165812	3.873950	-3.136010
8.C	-2.537712	4.136839	-2.631717
9.C	-3.212200	-4.874625	-2.125664
10.C	1.792544	-2.170454	-2.660559
11.C	0.399850	2.775695	-2.370451
12.C	-3.005784	-1.137386	-2.171649
13.C	-1.802712	-4.572636	-1.576916
14.C	5.967883	0.661303	-1.422162

15.C	0.888326	-3.000894	-1.728185
16.C	1.487229	-4.410172	-1.546724
17.C	-4.068531	-1.085553	-1.079421
18.C	6.202590	-0.653819	-0.916584
19.C	6.393689	1.591492	-0.406062
20.C	-4.156751	1.296508	-0.442651
21.C	-0.591773	4.894132	-0.331254
22.C	-1.805713	-4.646263	-0.040762
23.C	2.409260	0.255924	-0.260815
24.C	6.800112	-0.540148	0.389865
25.C	-3.160971	2.326339	0.083392
26.C	6.929894	0.846136	0.697158
27.C	0.650825	4.559163	0.513188
28.C	3.168884	1.436225	0.344034
29.C	-1.714987	5.447666	0.568833
30.C	3.253508	-0.806277	0.439172
31.C	-4.195284	-0.439891	1.290552
32.C	3.637272	1.107178	1.660110
33.C	3.688457	-0.330075	1.722037
34.C	-3.206064	-1.381822	1.965356
35.C	0.729161	-2.900619	2.843262
36.C	-0.135486	1.953628	3.430677
37.C	0.335866	0.493907	3.578840
38.C	-0.293457	-2.537968	3.933975
39.C	-1.333449	-3.664746	4.091988
40.C	1.340123	0.374082	4.743388
41.C	-3.197919	0.778509	4.930277
42.C	-2.378103	-0.521893	5.038801
43.C	-1.776613	-0.660177	6.453777
44.H	-1.184682	-1.817288	-6.002437
45.H	-0.793919	-4.322319	-5.902055
46.H	0.191793	-1.518023	-4.926480
47.H	0.563379	-4.180202	-4.771106
48.H	-1.399791	-0.862861	-4.519134
49.H	-2.183442	3.126665	-4.547170
50.H	-0.830116	-5.199708	-4.359087
51.H	-2.310416	-3.190820	-4.297951
52.H	-3.884767	3.521305	-4.252405
53.H	-3.094298	5.900061	-3.808015
54.H	-0.438155	1.806688	-4.160008
55.H	0.592666	4.237488	-4.003000
56.H	-1.361384	5.532423	-3.868421
57.H	-3.232107	-4.935656	-3.223350
58.H	2.123431	3.485630	-3.523149
59.H	1.129853	1.160979	-3.653728
60.H	-3.125964	2.151706	-3.407887
61.H	1.877601	-2.638930	-3.652843
62.H	-3.344257	-1.864488	-2.929448
63.H	-2.058105	6.284242	-2.419948
64.H	-0.336087	0.721516	-2.767006
65.H	1.413976	-1.149908	-2.801431
66.H	-2.983359	-0.164178	-2.697204

67.H	1.395556	4.744890	-2.505216
68.H	5.542037	0.913793	-2.388363
69.H	-3.943900	-4.108131	-1.823119
70.H	-3.584848	-5.838832	-1.738400
71.H	-3.419797	4.255017	-1.972359
72.H	1.485960	-4.976174	-2.491306
73.H	2.812959	-2.093079	-2.250431
74.H	-1.138400	-5.379812	-1.941798
75.H	-5.075247	-0.877713	-1.492932
76.H	1.044522	2.434963	-1.539375
77.H	5.967828	-1.583656	-1.426253
78.H	-4.174601	1.360048	-1.538016
79.H	2.581166	0.222200	-1.346914
80.H	-0.308311	5.700874	-1.033964
81.H	2.537477	-4.342560	-1.214313
82.H	6.333040	2.674250	-0.471105
83.H	-4.104579	-2.064211	-0.583039
84.H	0.947638	-5.009863	-0.801251
85.H	0.907757	-2.503213	-0.737542
86.H	1.527312	4.337308	-0.112119
87.H	-5.185120	1.480607	-0.074207
88.H	-3.559490	3.330233	-0.137035
89.H	-2.158277	-5.629530	0.314594
90.H	-2.612767	5.732031	-0.000958
91.H	3.065212	2.459000	-0.014111
92.H	-2.476118	-3.886314	0.388172
93.H	3.218007	-1.862019	0.175212
94.H	-0.811344	-4.476794	0.394786
95.H	7.087561	-1.369732	1.029168
96.H	0.921697	5.404214	1.169772
97.H	-1.375862	6.345359	1.113574
98.H	0.478129	3.684441	1.156525
99.H	7.335695	1.265181	1.612959
100.H	-5.211303	-0.878079	1.229581
101.H	-3.133366	2.268032	1.187907
102.H	-2.019612	4.708911	1.325595
103.H	-3.179666	-2.337150	1.410470
104.H	-4.261233	0.478960	1.888098
105.H	0.242168	-2.966885	1.857398
106.H	3.929562	1.798718	2.447206
107.H	-0.841534	2.090041	2.596788
108.H	4.026888	-0.927534	2.565615
109.H	0.891468	0.244815	2.650815
110.H	1.533323	-2.156460	2.756957
111.H	-3.615449	-1.635473	2.957859
112.H	1.196950	-3.880456	3.041408
113.H	0.721280	2.621133	3.244154
114.H	-1.916251	-3.802579	3.168252
115.H	-0.637408	2.315898	4.340575
116.H	-3.635271	0.914821	3.930992
117.H	-0.842293	-4.628861	4.308726
118.H	2.190650	1.060224	4.593092

119.H	-2.043865	-3.469116	4.908812
120.H	0.253344	-2.452593	4.892446
121.H	1.751107	-0.641118	4.840621
122.H	-3.091337	-1.360878	4.924415
123.H	-2.578105	1.664004	5.135192
124.H	-4.024858	0.786257	5.660863
125.H	0.880200	0.637824	5.708430
126.H	-1.227635	-1.604214	6.586594
127.H	-1.080352	0.162423	6.679276
128.H	-2.570633	-0.630267	7.220086
129.Co	4.913091	0.372341	0.328774
130.N	-1.675208	-1.467985	-1.608647
131.N	-1.820749	2.089714	-0.502280
132.N	-3.707881	-0.067610	-0.060576
133.N	0.959491	0.189554	-0.038189
134.N	-1.869330	-0.759067	2.031212
135.Si	-0.966603	-2.960413	-2.258528
136.Si	-1.160111	3.429314	-1.456910
137.Si	-1.080835	-0.802836	3.623066
138.U	-0.986410	0.008523	0.031565

Energy: −764.06220827 eV

Table S7. Final coordinates and single point energies of 4' and 4''

C	1.55303	-3.69408	-5.23879
C	0.63913	-2.73294	-4.47056
C	-0.79651	-3.30047	-4.42120
C	3.71144	-0.95524	-3.84663
C	-0.86684	0.61067	-3.85198
C	0.59428	0.35519	-3.48477
C	3.21199	-2.07728	-2.93163
C	1.74226	4.69906	-2.74599
C	-1.48527	2.64616	-2.63101
C	-2.95866	0.69022	-2.58466
C	1.46084	-4.78458	-1.66546
C	-2.99088	-0.56814	-1.75699
C	3.88794	-1.94597	-1.55284
C	1.26346	5.12676	-1.36595
C	0.91376	-3.35528	-1.35863
C	-1.37784	3.10747	-1.20834
C	2.17859	6.23808	-0.88794
C	3.20000	1.96490	-1.11582
C	-0.53408	-3.45065	-0.94729
C	-6.00052	0.91098	-0.53377
C	2.67357	2.69953	0.10767
C	-4.94909	1.21516	0.54449
C	3.81252	3.50325	0.74341
C	-4.68216	-1.85988	0.84035
C	-3.73410	-3.07821	1.02535
C	-0.75076	5.07486	1.58158
C	0.67433	4.49017	1.55415

C	-5.87680	-1.98391	1.78902
C	-5.61952	1.77600	1.80419
C	3.62128	-0.32046	1.81592
C	4.01135	-1.63416	2.07774
C	-2.78363	0.05305	2.46146
C	0.80230	-3.58977	2.60033
C	-2.12378	1.44806	2.53998
C	-0.25310	-2.63560	2.65673
C	0.84524	3.52891	2.74222
C	3.23739	0.25567	3.02566
C	3.98524	-1.80193	3.46183
C	-3.47208	-0.29173	3.72487
C	1.50130	-3.52504	3.83652
C	-0.11279	-1.93079	3.84367
C	3.45053	-0.68593	4.05494
C	0.95154	-2.49755	4.56764
H	1.18154	-3.86280	-6.10637
H	2.42275	-3.29868	-5.33493
H	-1.11872	-3.42974	-5.31661
H	0.58305	-1.92323	-5.02046
H	1.62856	-4.51864	-4.75612
H	3.35211	-1.07761	-4.72717
H	-0.92034	1.24322	-4.58476
H	1.06984	-0.00372	-4.25148
H	-1.28971	-0.21730	-4.13113
H	-0.79238	-4.14261	-3.95881
H	4.67005	-0.97850	-3.88493
H	-1.36945	-2.68449	-3.96211
H	1.78271	5.46653	-3.32015
H	3.42469	-0.10771	-3.49733
H	-3.30001	0.51255	-3.47535
H	3.51001	-2.92416	-3.32386
H	1.13113	4.05601	-3.11026
H	1.02022	1.18654	-3.22412
H	-0.71279	2.94827	-3.13395
H	-2.27986	3.02167	-3.03996
H	2.61652	4.30724	-2.67402
H	0.95189	-5.17466	-2.37676
H	-3.51388	1.37385	-2.17419
H	-2.44611	-1.24847	-2.17989
H	2.38300	-4.72298	-1.92552
H	2.39186	6.81545	-1.62337
H	3.47490	2.60237	-1.77816
H	4.82769	-1.79132	-1.67013
H	-3.90073	-0.89941	-1.70616
H	-1.03998	-3.86180	-1.65084
H	0.37339	5.51863	-1.47985
H	-5.56471	0.58195	-1.32290
H	2.50838	1.40775	-1.47555
H	-1.34557	4.07683	-1.17915
H	3.75696	-2.75633	-1.05399
H	3.49976	-1.20890	-1.07504

H	-6.48273	1.71339	-0.74311
H	1.38567	-5.33018	-0.87949
H	3.95023	1.42171	-0.86270
H	2.98628	5.85756	-0.53370
H	-0.87826	-2.57270	-0.77710
H	-2.15666	2.81459	-0.70776
H	1.40501	-3.03181	-0.57266
H	1.73642	6.74301	-0.20128
H	-6.61212	0.24688	-0.20393
H	-5.05067	-1.94453	-0.06326
H	-0.60656	-3.98201	-0.15040
H	4.17987	4.10729	0.09503
H	-4.40592	1.94681	0.18440
H	-2.97751	-2.98660	0.44320
H	-4.20697	-3.88656	0.81026
H	-0.81520	5.79360	0.94888
H	2.45766	1.99792	0.75686
H	4.49910	2.90215	1.04495
H	3.61813	0.09794	0.98567
H	-6.38430	-2.76775	1.56418
H	-6.12535	2.55865	1.57405
H	-1.38263	4.38917	1.35495
H	3.47373	4.00143	1.48999
H	4.24517	-2.27601	1.44836
H	-6.43572	-1.20796	1.70491
H	1.29927	5.23397	1.68270
H	-1.60262	1.60027	1.74819
H	0.99806	-4.15360	1.88592
H	-3.43590	-3.11555	1.93638
H	-6.20566	1.11461	2.17663
H	-0.91435	-2.50849	2.01666
H	-0.94371	5.40708	2.46180
H	-4.94589	2.00711	2.44915
H	-5.56367	-2.05502	2.69330
H	-2.02846	-0.57187	2.41887
H	-2.80442	2.12051	2.61019
H	0.34798	2.72296	2.57637
H	1.77410	3.31366	2.85004
H	2.89932	1.11395	3.13672
H	-1.55210	1.48890	3.31009
H	0.51805	3.94548	3.54360
H	-3.66099	-1.23230	3.74233
H	-4.29489	0.19992	3.78601
H	4.28442	-2.55713	3.91454
H	2.20250	-4.07492	4.10059
H	-0.63354	-1.20904	4.11618
H	-2.90918	-0.06497	4.46901
H	3.26612	-0.57315	4.95940
H	1.23784	-2.22245	5.40859
N	-1.55721	1.15213	-2.66279
N	0.63922	-0.61685	-2.35627
N	-0.13160	2.54110	-0.58371

N	-2.47903	-0.29530	-0.38392
N	0.87830	-0.61503	1.24068
Si	1.31735	-2.16058	-2.77518
Si	1.08460	3.68161	-0.11078
Si	-3.72172	-0.23643	0.84714
U	-0.27891	0.26615	-0.48978
V	1.69413	-1.41137	2.47529

Energy: −729.76182548 (**4'**) and −729.75175800 (**4''**) eV

References

1. D. M. King, F. Tuna, E. J. L. McInnes, J. McMaster, W. Lewis, A. J. Blake and S. T. Liddle, *Nat. Chem.,* 2013, **5**, 482-488.
2. CrysAlisPRO version 40.69, Oxford Diffraction /Agilent Technologies UK Ltd, Yarnton, England.
3. G. M. Sheldrick, *Acta Cryst. Sect. A*, 2008, **64**, 112-122.
4. A. L. Spek, *Acta Cryst. Sect. D*, 2009, **65**, 148-155.
5. G. M. Sheldrick, *Acta Cryst. Sect. C*, 2015, **71**, 3-8.
6. O. V. Dolomanov, L. J. Bourhis, R. J. Gildea, J. A. K. Howard and H. Puschmann, *J. Appl. Cryst.*, 2009, **42**, 339-341.
7. L. J. Farrugia, *J. Appl. Cryst.*, 2012, **45**, 849-854.
8. Persistence of Vision (TM) Raytracer; Persistence of Vision Pty. Ltd.: Williamstown, Victoria, Australia.
9. L. Chatelain, E. Louyriac, I. Douair, E. Lu, F. Tuna, A. J. Wooles, B. M. Gardner, L. Maron and S. T. Liddle, *Nat. Commun.*, 2020, **11**, 337.
10. C. Fonseca Guerra, J. G. Snijders, G. Te Velde and E. J. Baerends, *Theor. Chem. Acc.*, 1998, **99**, 391-403.
11. G. te Velde, F. M. Bickelhaupt, E. J. Baerends, C. Fonseca Guerra, S. J. A. van Gisbergen, J. G. Snijders and T. Ziegler, *J. Comput. Chem.*, 2001, **22**, 931-967.
12. E. Van Lenthe, E. J. Baerends and J. G. Snijders, *J. Chem. Phys.*, 1993, **99**, 4597-4610.
13. E. Van Lenthe, E. J. Baerends and J. G. Snijders, *J. Chem. Phys.*, 1994, **101**, 9783-9792.

14. E. Van Lenthe, A. E. Ehlers and E. J. Baerends, *J. Chem. Phys.*, 1999, **110**, 8943-8953.
15. S. H. Vosko, L. Wilk and M. Nusair, *Can. J. Phys.*, 1980, **58**, 1200-1211.
16. A. D. Becke, *Phys. Rev. A*, 1988, **38**, 3098.
17. J. P. Perdew, *Phys. Rev. B*, 1986, **33**, 8822.
18. E. D. Glendening, C. R. Landis, and F. Weinhold, *J. Comput. Chem.*, 2013, **34**, 2134-2134.
19. R. F. W. Bader, *Atoms in Molecules: A Quantum Theory*, Oxford University Press, New York, 1990.
20. R. F. W. Bader, *J. Phys. Chem. A*, 1998, **102**, 7314-7323.
21. L. C. Motta and J. Autschbach, *Nat. Commun.*, 2023, **14**, 4307.
22. G. Li Manni, I. Fdez. Galván, A. Alavi, F. Aleotti, F. Aquilante, J. Autschbach, D. Avagliano, A. Baiardi, J. J. Bao, S. Battaglia, L. Birnoschi, A. Blanco-González, S. I. Bokarev, R. Broer, R. Cacciari, P. B. Calio, R. K. Carlson, R. Carvalho Couto, L. Cerdán, L. F. Chibotaru, N. F. Chilton, J. R. Church, I. Conti, S. Coriani, J. Cuéllar-Zuquin, R. E. Daoud, N. Dattani, P. Decleva, C. De Graaf, M. G. Delcey, L. De Vico, W. Dobrautz, S. S. Dong, R. Feng, N. Ferré, M. Filatov(Gulak), L. Gagliardi, M. Garavelli, L. González, Y. Guan, M. Guo, M. R. Hennefarth, M. R. Hermes, C. E. Hoyer, M. Huix-Rotllant, V. K. Jaiswal, A. Kaiser, D. S. Kaliakin, M. Khamesian, D. S. King, V. Kochetov, M. Krośnicki, A. A. Kumaar, E. D. Larsson, S. Lehtola, M.-B. Lepetit, H. Lischka, P. López Ríos, M. Lundberg, D. Ma, S. Mai, P. Marquetand, I. C. D. Merritt, F. Montorsi, M. Mörchen, A. Nenov, V. H. A. Nguyen, Y. Nishimoto, M. S. Oakley, M. Olivucci, M. Oppel, D. Padula, R. Pandharkar, Q. M. Phung, F. Plasser, G. Raggi, E. Rebolini, M. Reiher, I. Rivalta, D. Roca-Sanjuán, T. Romig, A. A. Safari, A. Sánchez-Mansilla, A. M. Sand, I. Schapiro, T. R. Scott, J. Segarra-Martí, F. Segatta, D.-C. Sergentu, P. Sharma, R. Shepard, Y. Shu, J. K. Staab, T. P. Straatsma, L. K. Sørensen, B. N. C. Tenorio, D. G. Truhlar, L. Ungur, M. Vacher, V. Veryazov, T. A. Voß, O. Weser, D. Wu, X. Yang, D. Yarkony, C. Zhou, J. P. Zobel and R. Lindh, *J. Chem. Theory Comput.*, 2023, **19**, 6933–6991.

23. B. O. Roos, R. Lindh, P.-Å. Malmqvist, V. Veryazov and P.-O. Widmark, *J. Phys. Chem. A*, 2004, **108**, 2851–2858.

24. B. O. Roos, R. Lindh, P.-Å. Malmqvist, V. Veryazov and P.-O. Widmark, *Chem. Phys. Lett.*, 2005, **409**, 295–299.

25. B. O. Roos, R. Lindh, P.-Å. Malmqvist, V. Veryazov and P.-O. Widmark, *J. Phys. Chem. A*, 2005, **109**, 6575–6579.

26. M. Reiher, *Theor. Chem. Acc.*, 2006, **116**, 241–252.

27. *molcas_suite 1.32.0*, https://pypi.org/project/molcas-suite/, https://gitlab.com/chilton-group/molcas_suite

28. H. Eicher and F. H. Köhler, *Chem. Phys.*, 1988, **128**, 297-309.

29. M. Kauppa and F. H. Köhlerb, *Coord. Chem. Rev.*, 2009, **253**, 2376-2386.

30. T. A. Jackson, J. Krzystek, A. Ozarowski, G. B. Wijeratne, B. F. Wicker, D. J. Mindiola and J. Tesler. *Organometallics*, 2012, **31**, 8265–8274.
